# Performance measurement of ESG-themed megatrend investments in global equity markets using pure factor portfolios methodology

**DOI:** 10.1371/journal.pone.0244225

**Published:** 2020-12-22

**Authors:** Helena Naffa, Máté Fain

**Affiliations:** Department of Finance, Corvinus University of Budapest, Budapest, Hungary; University of Almeria, SPAIN

## Abstract

ESG factors are becoming mainstream in portfolio investment strategies, attracting increasing fund inflows from investors who are aligning their investment values to Sustainable Development Goals (SDG) declared by the United Nations Principles for Responsible Investments. Do investors sacrifice return for pursuing ESG-aligned megatrend goals? The study analyses the risk-adjusted financial performance of ESG-themed megatrend investment strategies in global equity markets. The analysis covers nine themes for the period 2015–2019: environmental megatrends covering energy efficiency, food security, and water scarcity; social megatrends covering ageing, millennials, and urbanisation; governance megatrends covered by cybersecurity, disruptive technologies, and robotics. We construct megatrend factor portfolios based on signalling theory and formulate a novel measure for stock megatrend exposure (MTE), based on the relative fund flows into the corresponding thematic ETFs. We apply pure factor portfolios methodology based on constrained WLS cross-sectional regressions to calculate Fama-French factor returns. Time-series regression rests on the generalised method of moments estimator (GMM) that uses robust distance instruments. Our findings show that each environmental megatrend, as well as the disruptive technologies megatrend, yielded positive and significant alphas relative to the passive strategy, although this outperformance becomes statistically insignificant in the Fama-French 5-factor model context. The important result is that most of the megatrend factor portfolios yielded significant non-negative alphas; which supports our assumption that megatrend investing strategy promotes SDGs while not sacrificing returns, even when accounting for transaction costs up to 50bps/annum. Higher transaction costs, as is the case for some of these ETFs with expense ratios reaching 80-100bps, may be an indication of two things: ESG-themed megatrend investors were willing to sacrifice ca. 30-50bps of annual return to remain aligned with sustainability targets, or that expense ratio may well decline in the future.

## 1. Introduction

Sustainable investing has become an attractive strategy both for investors and policymakers all around the world. According to the *Global Sustainable Investment Alliance’s 2018* report, sustainable investing reached $30.7 trillion at the start of 2018, a 34 per cent increase in two years. Also, the proportion of sustainable investments relative to total managed assets made up 33 per cent in 2018 while it was 21 per cent in 2012, which corresponds to an almost 60 per cent increase in six years [1]. Nevertheless, due to the lack of consistent definitions, it is difficult to determine the actual size of sustainable finance worldwide; for instance, *J*.*P*. *Morgan* estimates ‘only’ $3 trillion [2]. *United Nations’* 2030 Agenda for Sustainable Development sets out 17 Sustainable Development Goals (SDGs) and 169 targets which are to balance the economic, social and environmental dimensions of sustainable development [3–5]. Some of the goals are as follows: end hunger, achieve food security (SDG2), ensure healthy lives and promote well-being for all at all ages (SDG3), make cities and human settlements inclusive, safe, resilient and sustainable (SDG11).

Sustainable investing has at least 50 years of history, as the first related publications of *Moskowitz*, *Bragdon and Marlin*, *Bowman and Haire*, *Belkaoui* [6–9] appeared in the ‘70s. However, the concept of sustainable investing covers numerous different strategies and approaches; besides, several alternative names and terms exist as well. This heterogeneity in both terminology and investment strategies are apt to give rise to misunderstandings among academics and practitioners [10, 11]. For simplicity, we use the widely accepted terms of responsible investing (RI), sustainable investing (SI), socially responsible investing (SRI), environmental-social-governance (ESG) investing interchangeably throughout the study.

Further, according to *GSIA* [12], there are seven representative ESG investing strategies: exclusionary screening, best-in-class screening, norm-based screening, ESG integration, sustainability-themed investing, impact/community investing, and corporate engagement. Sustainability-themed ESG investment strategies are in the focus of our paper. Based on *UNCTAD* definition [13], ESG-themed portfolios include stocks that only concentrate on one particular sustainability theme (for example, gender equality or low carbon). However, stocks also belong to this group if they primarily focus on only one ESG pillar (environment, social or governance); alternatively, they track a ‘quasi sector’, such as energy efficiency or food security. We also introduce the term ‘megatrend’ as a closely related concept. *Naisbitt* and *Boesl-Bode* define megatrends as large transformative social, environmental, economic, political, and technological changes that could dramatically alter daily life [14, 15].

Sustainability themed investing approach is among the youngest ESG strategies, given that at the end of 2012, only $70 billion had been invested in ESG-themed funds. Since then, the strategy has shown impressive growth, with total Assets Under Management (AUM) reaching $1,018 million by the end of 2018. This figure corresponds to 56.23 per cent CAGR [1]. *UNCTAD*, referring to Blackrock, predicts that the ESG ETF market will exceed $500 billion by 2030 [13].

We analyse the following nine ESG-themed megatrends in the empirical section: energy efficiency, food security, water scarcity (environmental megatrends); ageing, millennials, urbanisation (social megatrends); cybersecurity, disruptive technologies, robotics (governance megatrends). The stocks in each thematic portfolio come from ESG-themed ETFs. Our stock selection approach relies on signalling theory meaning that the relative amount of money inflows targeting megatrend funds signal the portfolio management industry’s belief in those stocks being the best candidates to represent megatrends.

The research question of our paper is to examine whether megatrend investing is valid; that is, we test if megatrend factor portfolios could generate superior returns, on a risk-adjusted basis. We first compare the returns to the passive strategy (viz., we calculate CAPM alphas and Sharpe ratios relative to the market benchmark), and then measure the alpha applying various Fama-French model specifications (e.g. FF three-factor model, FF five-factor model). Our research question can also be interpreted as a test of the efficient market hypothesis (EMH) [16]. We also attempt to infer whether investing in megatrends may help in achieving some of the United Nations’ Sustainable Development Goals (SDGs) [3]. For the complete list of the SDGs see S1 Appendix in S2 File.

Our investment universe covers global equity markets spanning January 2015 and June 2019, which is a relatively short timeframe; however, the inflows into ESG-themed funds, as mentioned above, do not have a long history, therefore limiting our reference period. Further, there are studies in the corresponding literature on mutual fund performance that have a similarly shorter timeframe [17–19]. We source weekly trading data from Bloomberg and select the widely tracked MSCI All Country World Index (MSCI ACWI) as a benchmark. Besides ESG factors, we define eleven traditional style factors (beta, value, momentum, size, volatility, liquidity, profitability, growth, investment, leverage, and earnings variability) derived from 28 firm characteristics; 24 industry group factors (based on MSCI’s global industry classification standards, GICS); and 48 individual country factors to control for secondary factors. Altogether, we compiled a uniquely organised database, that includes approximately 15 million data points, covering roughly 2,700 individual stocks, for a period spanning 234 weeks, and measuring 106 factors.

A suitable methodology is required to capture the actual performance characteristics of the megatrend portfolios. Secondary factor exposures such as size, value, momentum or any other factors, could have a substantial consequence on the performance, i.e. these disturbing effects should be disentangled. To this end, we construct pure factor portfolios which rest on constrained WLS (CWLS) cross-sectional regressions. The cross-sectional calculations originate from the classic work of *Fama-MacBeth* [20, 21], and it is also in line with current empirical asset pricing literature [22–27]. Filtering out the effects of secondary factors is consistent with the creation of factor-mimicking long-short dollar-neutral portfolios. Concurrently, we avoid the usage of the ‘cumbersome’ double-sort quintile portfolio selection methodology introduced by *Fama and French* [28–30]. Next, we analyse the time series of megatrend portfolios’ returns resulting from CWLS by employing OLS with Newey-West standard errors. We also apply a GMM estimator that relies on a new and innovative set of distance instrumental variables (GMM-IV_d_) to account for the well-known phenomenon that the FF factors usually incorporate different forms of endogeneity [31–35].

The remainder of the paper is organised as follows. In the second section, we introduce the ESG literature, which is followed by a brief insight into the ‘ESG-themed megatrends’ concept. Next, we highlight the essential features of pure factor portfolios and the GMM-IV approach. The megatrend portfolio construction technique is also presented in this section. Subsequently, we introduce the unique database compiled for the empirical analysis. Finally, we present our empirical findings. The paper ends with a conclusion.

## 2. Literature review

There are many competing terms and definitions of sustainable investing. According to *Daugaard*, in the early times, the term ‘ethical’ was the commonly used expression. ‘Ethical’ was then replaced by ‘socially responsible investing’ (SRI). However, the relevance of ‘social’ had become controversial and was frequently replaced with the term ‘sustainable’ or researchers simply negligeed it; hence only the concept of ‘responsible investing’ (RI) remained [10]. Nowadays, ‘ESG’ is also applied routinely. We do not wish to make distinctions between these terms; therefore, we use them interchangeably throughout the text.

Sustainable investing has a rich literature that dates back to the early 1970s. The pioneering study of *Moskowitz* argues that responsible corporate behaviour might manifest in superior financial performance [6]. The influence of Moskowitz’s work is incontestable; as evidenced by the fact that the US Social Investment Forum has awarded the Moskowitz prize named in his honour since 1996, for the best article about the financial impact of socially responsible investing [10, 36]. In contrast to Moskowitz, *Friedman* claims that including ESG criteria in managerial decisions generates additional costs which, in turn, results in weaker financial performance [37]. These two contradictory views, supplemented by a third one on neutrality, have persisted until today and fundamentally determine research initiatives.

As mentioned, there exist three competing hypotheses in the management literature. The first one accepts the views of Moskowitz and emphasises the positive relationship between ESG and financial performance. Various management theories underpin this concept. Stakeholder theory [38–41] or good management theory [42] argue that the satisfaction of primary stakeholders (e.g. customers, employees, local communities, shareholders, natural environment) is critical in achieving superior financial performance. The second hypothesis argues for a negative relationship; namely, higher ESG performance lowers financial performance. The trade-off hypothesis [37, 43–46] declares that higher ESG performance is expensive: resource reallocation to socially responsible activities like charity, community development do not pay off [43], but higher operating costs are incurred due to internalisation of externalities [46]. The third hypothesis is the ‘no effect’ premise, which is often attributed to *McWilliams and Siegel* [47, 48]. The authors claim that incorporating R&D factors in the analysis of the ESG and financial performance relationship eliminate the positive impact, resulting in neutrality.

Over the past fifty years, a tremendous number of studies have been culminated examining the actual relationship between ESG and financial performance. Further, parallel with primary researches, several summarising literature reviews have also been published [49–53]. Probably the most comprehensive one is written by *Friede*, *Busch and Bassen* [54] who combine the findings of about 2,200 individual papers using second-order meta-analysis and concluding that roughly 90 per cent of studies found a nonnegative ESG-financial performance relationship.

Our study aims to measure the *market performance* of ESG-themed investing. Though the ESG versus market performance relation is characterised by the same three hypotheses (neutral, positive, negative) as those emphasised in the management literature, some specific facets are worth mentioning. The no-effect hypothesis is closely related to the modern portfolio theory (MPT) of *Markowitz* [55] and the efficient market hypothesis (EMH) of *Fama* [16]. The former argues that there is no return premium for factors that bear only idiosyncratic risk, i.e. it is assumed that ESG risks can be diversified [56]. The latter maintains that stock prices reflect all available and relevant information; hence it is impossible to achieve superior risk-adjusted returns relative to the market portfolio [57].

Some equilibrium models support the ‘trade-off’ hypothesis [46, 58, 59]. Each suggests that socially responsible stocks have a lower cost of capital either due to incomplete information [58], investor preferences [59] or the internalisation of externalities [46] which, in turn, results in higher valuation and lower future (expected) return [60, 61]. Another critical view, according to *Bauer*, *Koedijk and Otten*, is that ESG investments are likely to underperform in the long run because ESG portfolios are by nature subset of the market portfolio, i.e. the degree of diversification is lower [56].

*Hamilton et al*. and *Renneboog et al*. claim that investors may do well while doing good; viz., investors earn positive risk-adjusted returns while contributing to a good cause [52, 62]. Outperformance happens if ESG screening procedures generate value-relevant information otherwise not available to investors. ‘Value-relevant information’ indicates that the ‘doing well while doing good’ hypothesis might hold if markets misprice social responsibility [56, 62]; therefore, it is against the EMH [52].

According to GSIA, ESG-themed investments are still in their infancy, but they have exceptional growth potential, which is also supported by the fact that they achieved and maintained a 56.23 per cent CAGR between 2012 and 2018. Due to its short history, to the best of our knowledge, only a limited number of studies have paid attention to ESG-themed (megatrend) investment strategies. *Alvarez and Rodríguez* [17] focused on the water sector, *Malladi* [63] constructed children-oriented indices, *Martí-Ballester* [5] analysed the performance of SDG mutual funds dedicated to biotechnology and healthcare sectors, while *Muley et al*. [18] evaluated thematic based infrastructure mutual fund schemes in India. Renewable energy and climate change themes are probably the most popular among scholars. *Ibikunle and Steffen* [64] measured European green mutual fund performance, *Reboredo et al*. [19] question if investors pay a premium for ‘going green’, *Martí-Ballester* [65–67] also analysed sustainable energy-related mutual funds. At the same time, *Dopierała*, *Mosionek-Schweda and Ilczuk* [68] test whether asset allocation policy affects the performance of climate-themed mutual funds in the Scandinavian markets (Denmark, Norway, and Sweden).

Our research contributes to the existing literature by analysing some less emphasised E-, S-, and especially G-themed investment strategies. Further, we apply a combination of pure factor portfolios construction technique and GMM-IV_d_ approach, which has not been employed in sustainable investment literature yet.

## 3. Megatrends

We analyse the following nine ESG-themed megatrend equity portfolios: energy efficiency, food security, water scarcity (environmental megatrends); ageing, millennials, urbanisation (social megatrends); cybersecurity, disruptive technologies, robotics (governance megatrends) (see Table 1).

**Table 1:** Megatrends and themes.

Megatrends	Themes
Environment (E)	Energy efficiency
	Food security
	Water scarcity
Social (S)	Ageing
	Millennials
	Urbanisation
Governance (G)	Disruptive technology
	Cybersecurity
	Robotics

Classifying technological megatrends such as cybersecurity, robotics as well as disruptive technologies as governance-related megatrends might not seem to be straightforward. However, *Basie von Solms and Rossouw von Solms* highlights that corporate boards are realising that protecting their companies in the cyberspace is, in fact, a corporate governance responsibility; consequently, they are accountable for the related cyber risks in their companies [69]. According to *Fenwick and Vermeulen*, disruptive technologies and robotics continue to facilitate and drive more dispersed forms of corporate organisation–what they call ‘community-driven corporate organisation and governance’ [70, pp. 2–3]. The authors also maintain that technological changes enhance ‘decentralisation and disintermediation’ of business organisations, i.e. these disrupt traditional hierarchical forms. Summing up, the G-themed megatrend portfolios include firms that provide technological solutions related to specific governance issues. Each megatrend portfolio can be considered as ‘quasi-sectors’ that, at the same time, address ESG concerns.

Besides technological G megatrends, we provide a summary of the investment policies of the E and S themes. *Energy efficiency* megatrend invests in companies that provide products and services enabling the evolution of a more sustainable energy sector (for instance, solar and wind energy). Current primary energy demand accounts for 7–9 per cent of GDP and it is expected to grow by at least 1/3 by 2035, hence energy efficiency standards are continuously rising. *Food security* megatrend focuses on companies that operate mainly in agribusinesses: agricultural equipment, agribusiness and protein, farming, safety inspection firms, health and wellness, waste reduction. *Water scarcity* megatrend tracks companies that create products to conserve and purify water for homes, businesses and industries since 750 million people do not have access to clean drinking water.

*Ageing* megatrend aims to track the performance of developed and emerging market companies exposed to the growing purchasing power of the ageing population. Older persons (60+) are expected to more than double from 841 million in 2013 to more than 2 billion by 2050. Typical industry sectors are healthcare, insurance, senior living. *Millennials* megatrend seeks to track the performance of companies that provide exposure to the millennial generation. Millennials are emerging as a new dominant economic force. They are the largest generation by workforce headcount in the US. Attractive sectors for millennials are accommodation, autos, finance, media, technology, and travel. *Urbanisation* megatrend has been designed to replicate, to the extent possible, the performance of energy, industrial, and utility stocks, i.e. mainly infrastructure companies. The world’s urban population is expected to surpass 6 billion by 2045; therefore, investments that include home-building, infrastructure construction, civil engineering, air/road transport, and utilities could have immense potential.

We rely on signalling theory to select stocks from ESG-themed ETFs and allocate them into thematic megatrend portfolios. According to *Spence*, signalling theory is about to explain how decision-makers interpret and react in case of incomplete and asymmetrically distributed information among parties to a particular transaction [71]. The theory has its foundation on the premise that one party (e.g. seller) has complete information while external parties (e.g. buyers), have to rely on what the seller wishes to share. *Bergh and Gibbons* [72] emphasise that one way for buyers to reduce their risks is to identify observable characteristics that affect the probability of the seller’s performance. Such a characteristic is known as a signal. *Spence* [73] defines a signal as activities and characteristics which are visible and convey information in a market. According to *Connelly et al*. [74], signals are proper to reduce information asymmetry. Further, they are a form of credible communication that transmits information from sellers to buyers [72].

In our analysis, ETF portfolio managers’ (i.e. sellers) stock selection practices indicate (signal) to investors and analysts (i.e. buyers) that the companies they have carefully chosen are suitable for megatrend investment. Consequently, the relative amount of money inflows into megatrend funds signals the market’s belief in those stocks being the best candidates to represent megatrends. Our signalling theory approach rests on the assumption that market participants (viz., ETF portfolio managers) intend to select stocks that do belong to the various ESG megatrends. Conversely, if the stocks are ‘conventional’ and the ESG megatrend flag is only used as a ‘buzzword’, we may come to a wrong conclusion on the megatrends’ market performance. *Revelli and Viviani* [66] also raise this problem, which is the well-known concept of ‘window-dressing’ [75, 76]. In the next section, we introduce the formula with which one can calculate a company’s exposure to a particular megatrend.

## 4. Methodology

As *Clarke et al*. [23, 77] argue, the performance measurement of investment strategies requires two phases. The first one is to implement cross-sectional analyses (not necessarily regressions) to calculate factor returns (for a comprehensive summary of various factor models see *Walter and Berlinger* [78]). Secondly, time-series analyses (again, not necessarily regressions, see [25]) are applied to estimate portfolio alphas and sensitivities to the predetermined set of factors. In the literature, the *Fama-French (FF)* [28–30] and the *Fama-MacBeth (FM)* [20, 21] procedures are the two most commonly employed approaches to attain factor returns. Our empirical analysis rests on FM; however, in the following paragraphs, we briefly compare the underlying ‘philosophy’ of the two methods, to justify our choice. Next, we introduce the mathematical background of the FM method and the innovative GMM-IV_d_ used for times series analysis. The section ends with the formula applied to calculate megatrend exposures.

The well-known portfolio sorting technique of FF is the dominant analysis tool in empirical asset pricing [79–84]. Despite several favourable properties such as simplicity or the lack of any required functional format, it also has some drawbacks. One is that extending the number of explanatory factors beyond a certain number makes the modelling cumbersome [23]. Another problematic issue is the quasi arbitrary choice of the number of securities in the top and bottom portfolios (i.e. quintiles, deciles), which results in the exclusion of many stocks; thus, valuable information is lost [85]. Further, Fama-French rebalances the portfolios underlying SMB, HML, RMW, CMA only annually (at the end of June), that is, the factors might rely on stale information [26, 86]. Finally, in their recent article, *Fama and French* [25, p. 1893] summarise the essence of the FF5 factor *time-series analysis* as follows: it optimises the loadings on factors that are, in fact, not themselves optimised.

The FM method applies regressions that correct most of the FF procedure’s drawbacks but introduces new ones. Firstly, it simultaneously controls for several secondary exposures which is indeed a crucial requirement. Next, it uses the whole investment universe, not just the top and bottom quantiles. Further, it rebalances the factor portfolios at the beginning of each period. The drawbacks are the following: it is parametric (requires a strict functional format), endogeneity problems may emerge (e.g. errors-in-variables, omitted variables), and microcaps as wells as influential observations could have a significant impact [87].

Turning to the method of time series analysis, *Fama and French* emphasise four approaches of applying the output of cross-sectional analyses (either FF or FM) to explain market anomalies [25]. The first one (*I*.) is the traditional FF modelling technique using time-series (TS) FF factors (i.e. SMB, HML, RMW, CMA) in time-series regressions. The second (*II*.) is to apply cross-sectional (CS) FM factor returns in time-series regressions. The next approach (*III*.) is about ‘stacking’ FM (CS) regressions across periods (*t*); thus it becomes an asset pricing model (model, not regression) that can be used in time-series applications. Finally, the application of an approach that augments the FF TS modelling procedure with interaction variables that allow loadings for SMB, HML, RMW, and CMA to vary with the corresponding firm characteristics of FM (*IV*.).

In the empirical section of this study, we use the second approach (*II*.) to analyse the risk-adjusted performance of ESG-themed megatrend factor portfolios. *Back et al*. [26] applied (*II*.) and found that it explains five market anomalies out of thirteen, while FF (i.e. *I*.) was not able to clarify any of them. *Fama-French* [25] evaluate the performance of the four modelling techniques and argue that (*II*.) performs a bit better than (*I*.). However, the authors contend that (*III*.) provide a better description of returns than the other ones. Nevertheless, it is worth keeping in mind the remark by *Back et al*. [26, p. 4] that in the absence of an accepted theory explaining why there are risk premia associated with size, value, profitability, and investment; there cannot be a universally best method to define factors based on these characteristics.

### 4.1. Pure factor portfolios

We apply constrained multivariate cross-sectional regression analysis; specifically, we create pure factor portfolios (PFPs). The methodological details presented in this section can be found, inter alia, in [22–24, 77, 88, 89]. Furthermore, cross-sectional regressions are the basis of fundamental equity risk factor models provided by firms such as Axioma, Bloomberg, and MSCI [77]. In our analysis, the applied factors and firm characteristics rest on the factors of the Bloomberg fundamental factor model (see [90]), although we introduce some minor modifications.

Pure factor portfolios have the advantage of removing secondary factor effects without having a ‘black box’ nature of portfolio construction. Filtering out secondary factor exposures and isolating the effects of ESG factors, as mentioned above, is a crucial methodological requirement [91]. *Galema et al*. show that the book-to-market factor of the Fama-French model could incorporate some of the ESG characteristics [61]. In the 1980s, *Grossman and Sharpe* also found that the positive market-relative performance of the South Africa-free portfolios can be attributed to small firm size effect [92].

In the upcoming paragraphs, we first outline the original FM procedure briefly, then the mathematical background of our extended FM approach. Finally, we compare the two methods. The following mathematical derivation of the FM method and supplemental explanations can be found, among others, in *Fama* [21, Chapter 9, pp. 326–329], *Fama-French* [25], *Cochrane* [93], and *Back et al*. [26, 27]. The FM estimator is calculated by running cross-sectional regressions at each moment in time. With matrix algebra notation:

$$R_{t+1}=Z_{t}{\hat{F}}_{t+1}+u_{t+1},$$ (1)

where *R*_*t+1*_ is the (*N x 1*) vector of stock returns on *N* individual securities from *t* to *t+1*; *Z*_*t*_ is the (*N x K*) matrix of standardised firm characteristics at date *t* (z-scores), with a vector of ones as its first column; ${\hat{F}}_{t+1}$ is the (*K x 1*) vector of the ordinary least squares (OLS) values of the regression coefficients at *t+1*, and *u*_*t+1*_ is the (*N x 1*) vector of security return disturbances for *t+1* (*K* is the number of explanatory variables, including the market).

The OLS values for the regression coefficients are as follows:

$${\hat{F}}_{t+1}={\left({Z\prime }_{t}Z_{t}\right)}^{-1}{Z\prime }_{t}R_{t+1}$$ (2)

Note that the individual security weights in each factor portfolio are the elements of matrix *W*_*t*_:

$$W_{t}≝{\left({Z\prime }_{t}Z_{t}\right)}^{-1}{Z\prime }_{t}$$ (3)

One must emphasise that the portfolio weights are observable at *t*, even though the returns, hence the slope coefficients (*F*) are not observable until *t + 1*.

To determine the properties of the slope coefficients, we study the properties of *Z*_*t*_. Note first that

$$W_{t}Z_{t}={\left(Z\prime_{t}Z_{t}\right)}^{-1}Z\prime_{t}Z_{t}=I_{t},$$ (4)

where *I*_*t*_ is the (*K x K*) identity matrix. Given (4) and the fact that the first column of *Z*_*t*_ is an (*N x 1*) vector of 1’s, the FM procedure has some notable features [25, p. 1892]. Firstly, the *F* coefficients for each variable in an FM cross-section regression is the *t+1* return on a portfolio of the left-hand-side assets with weights for the assets that set the month *t* portfolio exposure of that given variable to one and zero to other explanatory variables. Secondly, each FM slope portfolio requires zero net investment; that is, the short positions of the left-hand-side assets finance the long positions in other left-hand-side assets. Finally, the intercept is the month *t+1* return on a standard portfolio of the left-hand-side (LHS) assets with security weights that sum to one and zero out each explanatory variable. The intercept, which is the level return, is the month *t+1* return common to all assets and not captured by the regression explanatory variables.

From a mathematical-statistical perspective, *our* pure factor portfolios (viz., FM procedure) rest on constrained weighted least squares (CWLS) multivariate cross-sectional regressions, which we explain in more details below. It is worth mentioning, however, that there is also a practical reason to construct PFPs: the method is available now on Bloomberg terminals for portfolio managers around the world, who can apply it in their daily decision-making processes (see Factors to Watch (FTW) function, Pure factor returns tab in Bloomberg). Nevertheless, the function is limited to developed markets at the time of writing.

This paper aims to compare the performance of pure megatrend factor portfolios with the benchmark market index and other traditional FF factors to find out whether megatrend factors could outperform it. To this end, market returns, and pure factor portfolio returns are calculated. PFP return calculation rests on the following formula:

$${pr}_{t+1}=\sum_{i=1}^{N}{pw}_{nt}*r_{nt+1},$$ (5)

where *pr*_*t+1*_ is the return of the given PFP at *t+1*, *pw*_*nt*_ is the pure factor weight of security *n* at date *t*, and *r*_*nt+1*_ is the return of security *n* at *t+1*.

The construction of PFPs uses traditional investment styles such as value, momentum, size or industries and countries measured by dummy variables. Calculation of stock weights rests on multi-factor constrained WLS regressions. By calculating the weights, the given factor portfolio will have a unit exposure relative to the benchmark. Parallel, it has market-neutral exposures to all other styles, including industries and countries. Industry and country neutrality means that the pure style portfolio has the same industry and country structure as the benchmark. PFPs are ‘fully invested’ long-short factor mimicking portfolios. To sum up, the critical issue is to measure pure stock weights, *pw*_*nt*._

The starting point is to write the cross-sectional regression equation on stock returns (for convenience, we drop the ‘hat’ operator from now on):

$$r_{nt+1}=r_{Mt+1}+\sum_{s}z_{nst}f_{st+1}+\sum_{i}x_{nit}f_{it+1}+\sum_{s}x_{nct}f_{ct+1}+u_{nt+1},$$ (6)

where *r*_*nt+1*_ is the return of stock *n* at *t+1*, *r*_*Mt+1*_ is the return of the market factor at *t+1*, *z*_*nst*_ is the standardised exposure of stock *n* to style factor *s* at time *t*, *f*_*st+1*_ is the active (market-relative) return of the style factor at time *t*. Similarly, *x*_*nit*_ and *x*_*nct*_ are the exposures of stock *n* to industry *i* and country *c* at *t*; *f*_*it+1*_ and *f*_*ct+1*_ are active returns for industry *i* and country *c* at time *t*. The *u*_*nt+1*_ is unexplained by the factors and is termed idiosyncratic, or stock-specific. The stock-specific returns are assumed to be mutually uncorrelated, and uncorrelated with the model factors.

From (6) it is apparent that every stock has unit exposure to the market factor (i.e., this average return is ‘modified’ by the *f* active returns). By contrast, dummy variables represent the country and industry exposures: a stock has unit exposure to its industry and country, and zero exposures to all the others. Style factor exposures are standardised scores (z-scores), which have a capitalisation-weighted mean of zero and standard deviation of one (i.e. stocks with negative exposure score below the average of the market).

Weighted standardisation (see [23, 77]) should be used for the rescaling process of raw or prior style exposures (e.g. P/E ratios for the value factor). This procedure ensures the consistency between the weighting scheme of the benchmark and the weights used to rescale prior style factor exposures. Since our benchmark is the MSCI ACWI Index which is a cap-weighted index, we apply the market capitalisation-weighting scheme. The formula is as follows:

$$z_{ns}=\frac{x_{ns}-\sum_{n=1}^{N}w_{nM}*x_{ns}}{\sqrt{\sum_{n=1}^{N}w_{nM}*x_{ns}^{2}-{\left(\sum_{n=1}^{N}w_{nM}*x_{ns}\right)}^{2}}},$$ (7)

where *x*_*ns*_ is the prior exposure of security *n* to a particular style factor *s*, *w*_*nM*_ is the market weight of security *n*. In the numerator, we subtract the capitalisation-weighted average of the prior exposure of style *s* from the prior style exposure *x*_*ns*_, and then the difference is divided by the standard deviation of the prior exposure.

After introducing our cap-weighted standardisation convention, we return to (6), but now we use the more convenient matrix notation (note that (8) is the same as (1)):

$$R_{t+1}=Z_{t}F_{t+1}+u_{t+1},$$ (8)

where *R*_*t+1*_ is the (*N x 1)* vector of stock returns at time *t+1*, *Z*_*t*_ is the (*N x K)* standardised factor exposure matrix (using (7)), *F*_*t+1*_ is the (*K x 1)* vector of active factor returns, and *u*_*t+1*_ is the (*N x* 1) vector of unexplained residuals (the first element of vector *F* is the market return, which is, by definition, not an active return). *K* equals the total number of factors, including the market factor; hence, the first column of *Z*_*t*_ contains 1’s (exposures to the market factor). We label the exposure matrix with ‘*Z’*, although not all the values are standardised: market, industry and country factor exposures are 1 and (0, 1), respectively.

One must recognise two exact collinearities in our model as the sum of industry and country factor exposures give one each (i.e. identical to the market factor). In other words, only *K-2* variables are genuinely independent; therefore, we must impose two constraints to obtain the *Z* matrix have linearly independent columns (without constraints the regression cannot be solved as *(Z’Z)*^*-1*^ does not exist, i.e. it is singular). The sum of industry and country returns equals the market return; hence, the market-relative industry and country returns should equal zero. *Heston-Rouwenhorst* [94], *Menchero* [22] applied these equations to eliminate exact multicollinearity. The simple mathematical formulae are as follows (*w*_*it*_ and *w*_*ct*_ are the market capitalisations for each *i* industry and *c* country factor at time *t*):

$$\sum_{i}w_{it}f_{it+1}=0$$ (9)

$$\sum_{c}w_{ct}f_{ct+1}=0$$ (10)

We can write the constraints in matrix form:

$$F_{t+1}=C_{t}G_{t+1},$$ (11)

where *C*_*t*_ is the *K x (K—2)* constraint matrix at date *t*, and *G*_*t+1*_ is the *(K—2) x 1* vector of auxiliary returns in time *t+1*. Below, (12) is an example for *C*_*t*_*G*_*t+1*_. Here, for the sake of simplicity, four factors, including the market and three industry factors are involved; therefore, only one constraint applied:

$$\left[\begin{array}{c} r_{M}\\ f_{i1}\\ f_{i2}\\ f_{i3} \end{array}\right]=\left[\begin{array}{ccc} 1 & 0 & 0\\ 0 & 1 & 0\\ 0 & 0 & 1\\ 0 & w_{i1}/w_{i3} & w_{i2}/w_{i3} \end{array}\right]\left[\begin{array}{c} g_{M}\\ g_{i1}\\ g_{i2} \end{array}\right],$$ (12)

The heteroscedastic nature of the stock-specific returns (*u*_*t+1*_) and the influence of small stocks is well-known; therefore, weighted least squares (WLS) regressions ought to be applied. There are more technical opportunities to manage these challenges; therefore, we follow the work of *Clarke et al*. [77] when we use market capitalisation as weights. The authors argue that it is quite common to use equal weights and square-root-of-market-capitalisation-weights (many commercial risk-factor models use this), the latter, however, produces similar results to the market capitalisation weighting scheme.

We use the (*N x N*) *V*_*t*_ diagonal matrix in (13) and substitute *C*_*t*_*G*_*t+1*_ (11) for *F*_*t+1*_. The diagonal elements of *V*_*t*_ are the securities’ market capitalisations (*w*_*nM*_) at *t*:

$$V_{t}R_{t+1}=V_{t}Z_{t}C_{t}G_{t+1}+V_{t}u_{t+1},$$ (13)

Some changes in the variables make (13) a bit simpler (${\tilde{R}}_{t+1}$ *= V*_*t*_*R*_*t+1*_, *Y*_*t*_ *= V*_*t*_*Z*_*t*_*C*_*t*_ and ${\tilde{u}}_{t+1}$ = *V*_*t*_*u*_*t+1*_):

$${\tilde{R}}_{t+1}=Y_{t}G_{t+1}+{\tilde{u}}_{t+1}.$$ (14)

Now, in (14), we have the standard homoscedastic regression equation again. The OLS solution is as follows:

$$G_{t+1}={\left(Y_{t}\prime Y_{t}\right)}^{-1}Y_{t}\prime {\tilde{R}}_{t+1}.$$ (15)

Making substitutions to transform back the variables, we obtain the final solution:

$$F_{t+1}=C_{t}{\left(C_{t}\prime Z_{t}\prime V_{t}Z_{t}C_{t}\right)}^{-1}C_{t}\prime Z_{t}\prime V_{t}R_{t+1}.$$ (16)

In (17), we denote the *(K x N)* matrix of pure factor active weights of securities with *PW*_t_ (active weights mean, similarly to active returns, the weight of securities above or below the market weights, i.e. the over- or underweighting relative to the market):

$${PW}_{t}≝C_{t}{\left({C^{\prime }}_{t}{Z_{t}}^{\prime }V_{t}Z_{t}C_{t}\right)}^{-1}C_{t}\prime Z_{t}\prime V_{t}.$$ (17)

According to (17), the active security weights in PFPs can be calculated directly by using the cap-weighted standardisation procedure for firm characteristics based on (7). The product of pure security active weights, and the realised stock returns is the return of the pure factor portfolio in (5). The calculation of PFP returns can be derived alternatively, by using the slope coefficients (i.e. market return and factor portfolio active returns) in the CWLS cross-sectional regression of stock returns, *R*_*t+1*_, on standardised factor exposures, *Z*_*t*_, in Eqs (8) or, equivalently, in (16).

At last, we compare the weight matrices of the original (*W*_*t*_ in (3)) and the modified FM procedure (*PW*_*t*_ in (17)) to give a summary about the differences. The adapted CWLS regression has the following enhancement compared to the classical *Fama-MacBeth* regression technique (the explanations below, regarding the improvements, could be found in the studies of *Clarke et al*. [77, p. 16, and online appendix A] and *Menchero* [22]).

If one looks at the formulae, the first impression may be that (17) is more intricate; that is, it indeed considers issues that (3) does not. Firstly, the observations in each cross-sectional regression are weighted by market capitalisation, viz., we use the *V*_*t*_ diagonal matrix, which is missing in (3). Thus, including smaller stocks has little impact on the regression results, except that more missing or outlier values emerge among the explanatory variables. Secondly, each of the style and megatrend characteristics is shifted every period to have a cross-sectional capitalisation-weighted mean of zero. Together with observation weighting, this step makes the estimated regression intercept precisely equal to the return on a capitalisation-weighted portfolio of all admitted stocks. Non-zero values for the other four factors then measure exposures that are relative to the market portfolio. Further, every descriptor is scaled each period to have a cross-sectional standard deviation of one. In summary, (17) applies the “mean” and the “scale” adjustments for the firm characteristics based on capitalisation weighted standardisation of (7) while (3) uses arithmetic (i.e., equal-weighted) means and standard deviations for standardisation. Finally, we use constraints (*C*_*t*_) to manage exact multicollinearities, consequently, be capable of filtering out secondary industry and country exposures, which is not the case for the original FM method.

### 4.2. Time series analysis with GMM-IV_d_

After calculating PFP returns, the next step is measuring and testing the megatrend portfolios’ alphas using time-series regressions. In the empirical section, we test the traditional CAPM, the Fama-French-Carhart (FFC), the Fama-French 5 factor (FF5) and the augmented version of the FF5 factor model that includes liquidity as a sixth factor (FF5L). Eq (18) is the CAPM:

$${RP}_{et}=\alpha_{e}+b_{1e}{MRP}_{t}+u_{et},$$ (18)

where *RP*_*et*_ is the excess return (*R*_*et*_*—R*_*ft*_) of megatrend *e* at *t* (we use *e* as the abbreviation for ESG-themed megatrend); *R*_*ft*_ is the one-year US Treasury bill rate; *α*_*e*_ is the Jensen’s alpha; *MRP*_*t*_ is the market risk premium (*R*_*Mt*_*−R*_*ft*_) at *t*; *b*_*1e*_ is the beta of megatrend *e* (sensitivity to the market), and *u*_*et*_ is the error term. CAPM is an appropriate model for testing the performance of ESG-themed investments relative to the passive strategy.

Eq (19) is the FFC four-factor model:

$${RP}_{et}=\alpha_{e}+b_{1e}{MRP}_{t}+b_{2e}F_{SIZEt}+b_{3e}F_{VALUEt}+b_{4e}F_{MOMt}+u_{et},$$ (19)

where *F*_*SIZEt*_, *F*_*VALUEt*_, *F*_*MOMt*_ are, respectively, the market-relative pure factor returns from (16) for size, value, and momentum firm characteristics. The regression coefficients *b*_*2e*_, *b*_*3e*_, *b*_*4e*_ are the ESG-themed portfolios’ sensitivities to the prespecified factors. We employ the FFC model to account for the effects of momentum.

The next model is the FF5 factors:

$${RP}_{et}=\alpha_{e}+b_{1e}{MRP}_{t}+b_{2e}F_{SIZEt}+b_{3e}F_{VALUEt}+b_{4e}F_{PROITt}+b_{5e}F_{INVt}+u_{et},$$ (20)

where *F*_*PROFITt*_, *F*_*INVt*_ are PFP returns for profitability and investment factors. The coefficients *b*_*4e*_ and *b*_*5e*_ are the left-hand-side assets’ sensitivities to profitability and investment factors.

The last model is the FF5 augmented with a liquidity factor:

$${RP}_{et}=\alpha_{e}+b_{1e}{MRP}_{t}+b_{2e}F_{SIZEt}+b_{3e}F_{VALUEt}+b_{4e}F_{PROITt}+b_{5e}F_{INVt}+b_{6e}F_{LIQt}+u_{et},$$ (21)

In the empirical asset pricing literature, it is a common practice to identify new factors besides the traditional Fama-French exposures (*Cochrane* [95], thus, not inadvertently used the term ‘zoo of factors’). The effect of liquidity or illiquidity is a critical factor which is clearly in the focus of researchers (see *Amihud* [96], *Pástor and Stambaugh* [97] or *Racicot et al*. [98, 99]). Beyond liquidity, the applied factors are, in fact, very diverse: *López-García et al*. [100] applied a long term memory factor, *Chan et al*. [101] used R&D and advertising expenses, *Thomas and Zhang* [102] analysed the performance of inventory changes.

The critical methodological question is how to estimate the coefficients of each equation. We applied two methods: 1.) traditional OLS with *Newey-West* (HAC) standard errors [103], and 2.) generalised method of moments using innovative, robust distance instrumental variables (GMM-IV_d_), which can be found in *Racicot* [31], *Racicot et al*. [32, 104] and *Roy-Schijin* [35]. The GMM-IV_d_ method is suitable to address the various manifestations of endogeneity inherent in factor models [105]. According to *Racicot* [31], the GMM-IV_d_ approach provides solutions to measurement errors (errors-in-variables), specification errors.

Following *Racicot and Rentz* [104] the GMM estimator in (22) chooses the value, ${\hat{b}}_{e}$, that minimises a quadratic function of the moment conditions. We define the estimator as follow (we changed the notation of *Racicot and Rentz* slightly to have consistent formulae with the previous equations):

$${{\hat{b}}_{e}\equiv argmin}_{{\hat{b}}_{e}}\left\{T^{-1}{\left[d^{\prime }(RP-F{\hat{b}}_{e})\right]}^{\prime }WT^{-1}[d^{\prime }(RP-F{\hat{b}}_{e})]\right\}$$ (22)

The GMM-IV_d_ estimator makes the moment conditions as close to zero as possible. Each variable in (22) is defined below in (23) to (34). We start with *T*, which is the total number of observations (i.e. periods *t = 1*,*…*,*T*). *W* is a symmetric positive-definite matrix known as a weight matrix with the same number of rows and columns as the number of columns of *d*. We estimate W with the Newey-West HAC estimator. RP is defined as follow:

$$RP=F{\hat{b}}_{e}+u$$ (23)

where *F* is assumed to be an unobserved matrix of explanatory variables. The observed matrix of observed variables is assumed to be measured with normally distributed error:

$$F^{*}=F+v$$ (24)

${\hat{b}}_{e}$ is defined as:

$${\hat{b}}_{e}={\hat{b}}_{e2SLS}={\left(F\prime P_{Z}F\right)}^{-1}F\prime P_{Z}RE$$ (25)

*P*_*z*_ is defined as the standard ‘predicted value maker’ or ‘projection matrix’ used to compute:

$$P_{Z}=Z{\left(Z\prime Z\right)}^{-1}Z\prime ,$$ (26)

In (26), *Z* is the matrix of instruments (should not be confused with *Z* from section 4.1.). Here, Z is obtained by optimally combining the *Durbin* [106] and *Pal* [107] estimators using GLS.

Using the projection matrix, we can calculate the predicted values of *F*:

$$P_{Z}F=Z{\left(Z\prime Z\right)}^{-1}Z\prime F=Z\hat{\theta }=\hat{F}$$ (27)

From (27) extract the matrix of residuals

$$d=F-\hat{F}=F-P_{Z}F=(I-P_{Z})F$$ (28)

In (28) *d* is a matrix of instruments that can be defined *individually* in deviation form as

$$d_{it}=f_{it}-{\hat{f}}_{it}$$ (29)

As *Racicot* [31, p. 986] highlights (29) may be considered as a filtered version of the endogenous variables. It removes some of the nonlinearities embedded in the *f*_*it*_. Formula (29) is thus a smoothed version of *f*_*it*_ which might be regarded as a proxy for its long-term expected value, the relevant variables in the asset pricing models being theoretically defined on the explanatory variables’ expected values.

The next step is to calculate the values of ${\hat{f}}_{it}$ which is obtained by performing OLS regressions based on the *z* (cumulant) instruments:

$$f_{it}={\hat{\gamma }}_{0}+z\hat{\varphi }+\varsigma_{t}={\hat{f}}_{it}+\varsigma_{t}$$ (30)

(30) amounts to running a polynomial adjustment on each explanatory variable.

The z isntruments are defined as z = {z_0_,z_1_,z_2_}, where

$$z_{0}=\iota_{T}$$ (31)

$$z_{1}=f⨀f$$ (32)

$$z_{2}=f⨀f⨀f-3f[(D(f\prime f/T)]$$ (33)

$$D(f\prime f/T)=p\underset{T\to \infty }{\lim}(f\prime f/T)⨀I_{k}$$ (34)

In (31) *ι*_*T*_ stands for a vector of one (*T x 1*). In (32)–(34) *f* is the matrix of the explanatory variables expressed in deviation from their mean; the operator ⨀ is the Hadamard product; *D(f’f/T)* is a diagonal matrix and *I*_*k*_ is an identity matrix where *k* is the number of explanatory variables. Again, z_1_ contains the instruments used in the *Durbin* [106] estimator, and z_2_ contains the cumulant instruments employed by *Pal* [107]. Racicot and Rentz [103, p. 332] emphasise that the assumption of normality is a sufficient condition for the estimators to be consistent once measurement errors are purged using these third and fourth cross-sample moments as instruments for parameter estimation.

### 4.3. Calculating megatrend factor exposures

To quantify *megatrend exposures*, using dummy variables would seem an obvious solution: one could collect exchange-traded funds (ETFs) that consider themselves as thematic investment funds, then each company in these ETFs are classified into a particular megatrend, hence get a value of one. Those firms that are not listed in any of the thematic ETFs get a value of zero. In contrast, our idea is that megatrend exposures ought to be measured on a ratio scale as companies are different regarding how much they are affected by different megatrends, viz., how well they fit into megatrends. (Further, applying dummy variables would introduce another exact multicollinearity in our model, which is, in fact, not a real challenge to handle, but makes the modelling a bit more complicated.) The applied formula for megatrend exposures is, therefore, as follows:

$${MTE}_{nmt}=\frac{\sum_{e=1}^{E}{FI}_{nmt}}{{MCap}_{nt}},$$ (35)

where *MTE*_*nmt*_ is the megatrend exposure of stock *n* in megatrend *m* at time *t*. *FI*_*nmt*_ is the total fund inflow (the total number of share *n* multiplied by its stock price) into ETF *e* that invests in stock *n* and belongs to a particular megatrend *m* at time *t* (there are a total of *E* ETFs), and *MCap*_*nt*_ is the total market capitalisation of stock *n* at time *t*. The higher the ratio, the higher the exposure of a given stock to a particular megatrend *m*.

To have *FIs*, we analysed 37 ETFs that consider themselves as thematic funds (see them in S5 Appendix in S2 File). All the ETFs had more than $40 million AUM at the end of September 2019 (27.09.2019). The total AUM was $16,943 million. We should emphasise that, due to data limitations, we use constant positions (the number of stocks remains unchanged during the entire period, and reflects the status as of 20.09.2019.). Nevertheless, the stock prices vary weekly to quantify fund inflows for each week between 2015 and 2019.

## 5. Dataset

To obtain valid results, the sound choice of the investment universe is essential. According to *Cahan and Ji*, there are two types of security universes: coverage universe and estimation universe [90]. We employ a global investor perspective throughout this paper, that is, coverage universe includes theoretically ‘all’ the stocks that are traded in global markets. However, for practical reasons, a widely used index is satisfactory. We use the MSCI All Country World Index, which had more than 2.700 constituents in 2018. The estimation universe is the subset of stocks from the coverage universe used for constructing pure factor portfolios. The availability of critical variables such as stock price, total return and market capitalisation apart from standard data cleansing procedures determines the size of the estimation universe.

We collected weekly stock data from Bloomberg covering January 2015 and June 2019 on MSCI ACWI Index members to calculate total returns, nine megatrend exposures, 28 prior style descriptor exposures, 24 industry (based on second level GICS) and 48 country dummies. Prior style *descriptors* are the inputs to compute style *factor* exposures with principal component analysis (PCA). As a result of PCA, we get eleven style factors (see Table 2). (S2 Appendix in S2 File contains the detailed descriptions, calculation methods and applied Bloomberg codes related to each factor.)

**Table 2:** Pure style factors and factor-related descriptors.

Factor	Descriptor
Beta (B)	Market-relative beta: *Beta-1*
Value (V)	E/P
	CF/P
	BV/P
Momentum (M)	Return momentum
	Price momentum
	Sharpe-momentum
Size (S)	• ln(MCap)
	• ln(Assets)
	• ln(Sales)
Volatility (Vol)	Total volatility
	Residual volatility
	Price range
Liquidity (L)	*Amihud* liquidity ratio
Profitability (P)	ROE
	ROA
	ROIC/WACC
	Profit margin
Growth (G)	EBT growth
	Net income growth
	Sales growth
Investment (I)	Asset growth
Leverage (L)	Book leverage
	Market leverage
	Debts/Assets
Earnings variability (EV)	Sales variability
	Net income variability
	FCFF variability

Source: Own compilation based on Bloomberg’s US fundamental factor model [90].

All the stocks that were traded between 2015 and 2019 are analysed, which helps to eliminate survivorship bias. For precise statistical inference, we performed data cleansing procedures on a year-by-year basis. First, we excluded those companies that did not have, for any reasons, market price, total return or market capitalisation data. Second, the so-called penny stocks (stocks with a maximum price below five dollars) were removed (in line with [26, 87]).

Unfortunately, despite our best efforts, we had missing values for several descriptors and for many firms, which is not surprising as 28 company characteristics are analysed. S3 Appendix in S2 File presents the proportion of missing observations for each characteristic: one can see that 1.81 per cent of observations is missing which is relatively moderate (CF/P has the highest missing rate with 12.01 per cent); however, this represents 200–300 companies (i.e. many firms have only a few missing values). One solution could have been to delete these observations listwise; however, that would have decreased our sample size radically. Instead, we implemented multiple imputation (MI, [108]) procedures (we used Stata16). Due to the relatively low proportion of missing data, only three imputations were executed. We employed the Markov Chain Monte Carlo (MCMC) imputation procedure, and all the 28 descriptors were used.

The MCMC procedure assumes that all the variables in the imputation model have a joint multivariate normal distribution (MVN), probably the most common parametric approach for MI [109]. The specific algorithm used is called the data augmentation (DA) algorithm, which is an iterative MCMC procedure. The algorithm fills in missing data by drawing from a conditional distribution, in this case, an MVN, of the missing data given the observed data (for a detailed explanation of DA in Stata environment see [110]). In most cases, simulation studies have concluded that the assumption of MVN leads to reliable estimates even if the normality assumption is violated given sufficient sample size [111, 112]. Table 3 summarises the sample sizes year by year; hence the MCMC is an appropriate procedure for our analysis.

**Table 3:** Sample size after data cleansing and multiple imputation procedures.

Sample size	2015	2016	2017	2018	2019
MSCI ACWI members (30th June)	2 483	2 481	2 500	2 781	2 849
Companies in the final sample	1 915	1 893	1 953	2 031	2 040
Sample size/MSCI ACWI members	77.12%	76.30%	78.12%	73.03%	71.60%

Next, we specified winsorisation limits to ensure that extreme values would not affect statistical inferences (in line with [87]). The limits were the 1^st^ and the 99^th^ percentiles of each descriptor. We replaced each extreme descriptor value with the 1^st^ and the 99^th^ percentile.

**Fig 1:**
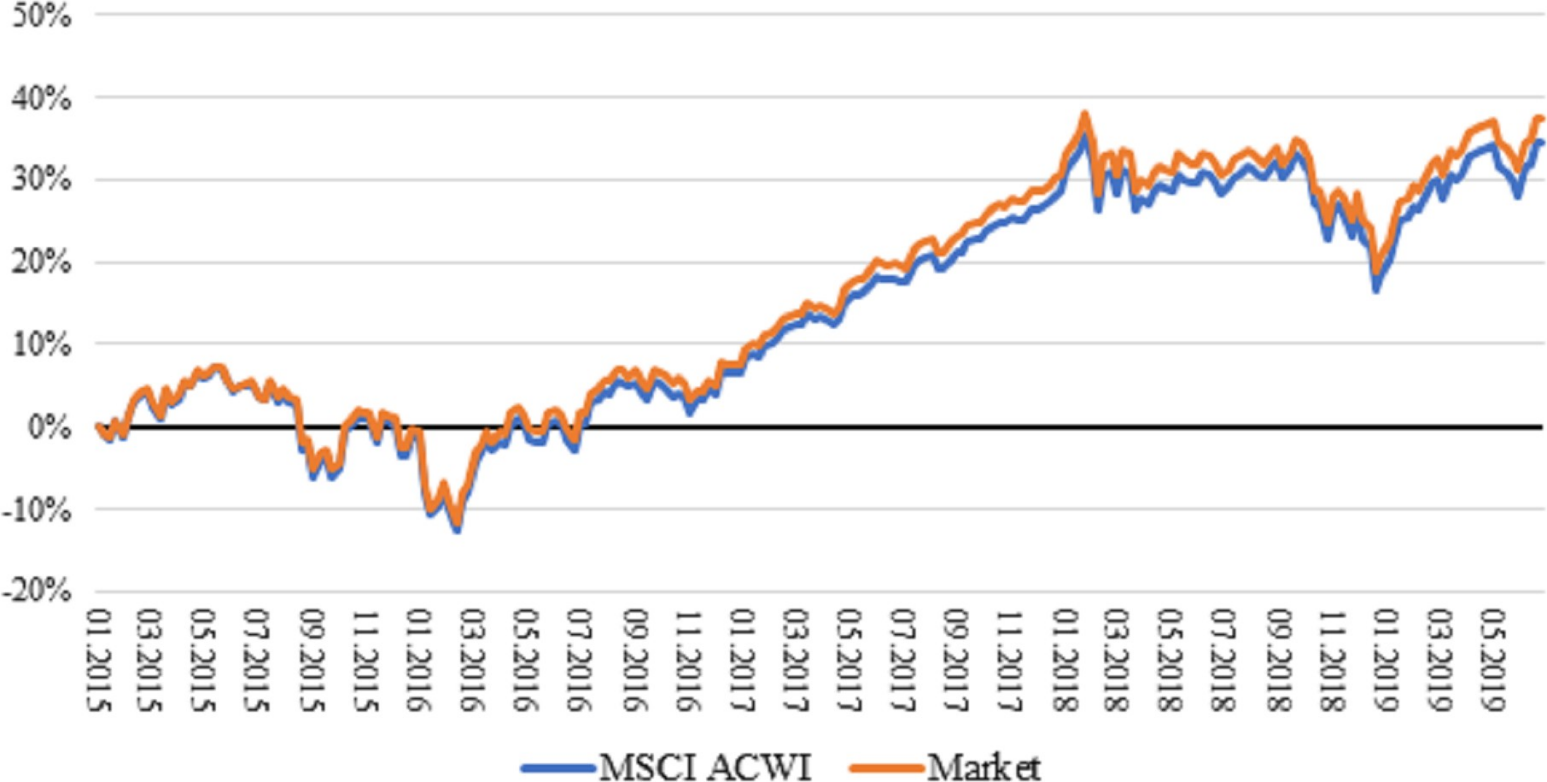
Cumulative total log return of MSCI ACWI Index and market portfolio. The market portfolio contains only companies that have prices, total returns and market capitalisations, and are not considered as penny stocks. “Market” portfolio is the reference point for the cross-sectional regressions. MSCI ACWI is the benchmark for the time series analysis.

The estimation universe covers on average 75 per cent of the benchmark, which we consider as sufficient. Due to consistency considerations, we construct a market-cap weighted portfolio (Market) of the estimation universe, which serves as the reference point in cross-sectional regressions. In Fig 1, one could see the cumulative total log-returns of MSCI ACWI and our ‘artificial’ Market portfolio. The prices move together and almost overlap each other (the cumulative return difference is 2.88 per cent for the entire period). It is good news since the created Market portfolio is the reference point to calculate active weights (and returns) of pure factor portfolios. The performance measurement of pure megatrend factor portfolios is measured relative to the benchmark index (MSCI ACWI).

After prior style descriptor calculations and data cleansing, as well as multiple imputation procedures, we use principal component analysis (PCA) for every week to calculate descriptor weights. The PCA results in the dimension reduction of descriptors. As a result of the PCA, we obtain eleven traditional style factors: market-relative beta, value, momentum, size, volatility, liquidity, profitability, growth, investment, leverage, and earnings variability.

The concept of market-relative beta hinges on the modified CAPM equation, and it is as follows:

$$R_{n}=R_{M}+{(\beta }_{n}-1)R_{M},$$ (36)

where *R*_*n*_ and *R*_*M*_ are the excess returns for stock *n* and the market, and (*β*_*n*_*—1*) is the market-relative beta. According to the traditional CAPM, the expected return on unscaled relative betas (i.e. before standardisation) should be equal to the market risk premium, which is the slope coefficient of the security market line (SML). When active returns are calculated (i.e. after standardisation) the return premium should be zero if CAPM assumptions hold. If the return premium is negative, the slope of SML is flatter or even downward sloping. Empirical researches [113] found that the SML is, in most of the time, flat or downward sloping (hence the name ‘low beta anomaly’). An alternative way of thinking about risk is in *Ormos-Zibriczky* [114]. The authors investigated entropy as a financial risk measure. Entropy explains the equity premium of securities with higher explanatory power than the classical beta parameter of the CAPM.

Turning back to Table 2, the value factor measures the cheapness of a particular stock with using the inverse P/E, P/CF and P/BV. The momentum factor combines three different metrics which are both standards in the academic literature and practice (for further details, see [115]). The size factor is the so-called ‘small-size’ factor, measured with negative logs. The Amihud ratio is the usual illiquidity ratio; however, we prefer measuring liquidity. Therefore, we calculated the inverse of it [116]. Earnings variability is the volatility of CF and P&L lines. Assets growth as an investment factor is in line with *Fama-French* [30].

Beyond style factors listed in Table 1, our empirical analysis also includes 48 *country and 24 industry group factors* (second level GICS) to neutralise their effects and to obtain pure megatrend (and style) factors (pure industry and country factors are style and megatrend neutral). We use dummy variables to measure sector and country factors (see S4 Appendix in S2 File)

## 6. Empirical results

Our research addresses the question if investing in ESG-themed megatrend equity factor portfolios could generate significant positive risk-adjusted returns. More formerly, we test the following two hypotheses.

*Hypothesis 1*: *Pure megatrend factor portfolios produced significant alphas*.

Statistically:

H_0_: α_e_ = 0
H_A_: α_e_ ≠ 0

Beyond measuring the Jensen’s alpha against the passive strategy (i.e. the test of (18)), we also test the difference of the Sharpe ratios to check the robustness of CAPM alpha. Our second hypothesis is the following:

*Hypothesis 2*: *The megatrend factor portfolios*, *based on the Sharpe ratios*, *produced significant risk-adjusted excess returns*, *relative to the passive strategy*.

Statistically:

H_0_: Sharpe-ratio (megatrend)—Sharpe-ratio (passive strategy) = 0
H_A_: Sharpe-ratio (megatrend)–Sharpe-ratio (passive strategy) ≠ 0

Sharpe ratio measures total risk (regarding the relation between total market risk and the volatility of various factors see the paper by *Csóka*, *Herings*, *and Kóczy* [117]). A general tool to test the significance of Sharpe ratios is the measure of *Jobson and Korkie* [118], which has been modified by *Memmel* [119]. Unfortunately, this test is not valid if returns are not normally distributed or have time-series nature (for a more detailed discussion about the possible mistakes and correct applications, see the comprehensive work of *Ledoit and Wolf* [120]). To solve this problem, we use HAC standard errors based on Newey-West.

**Fig 2:**
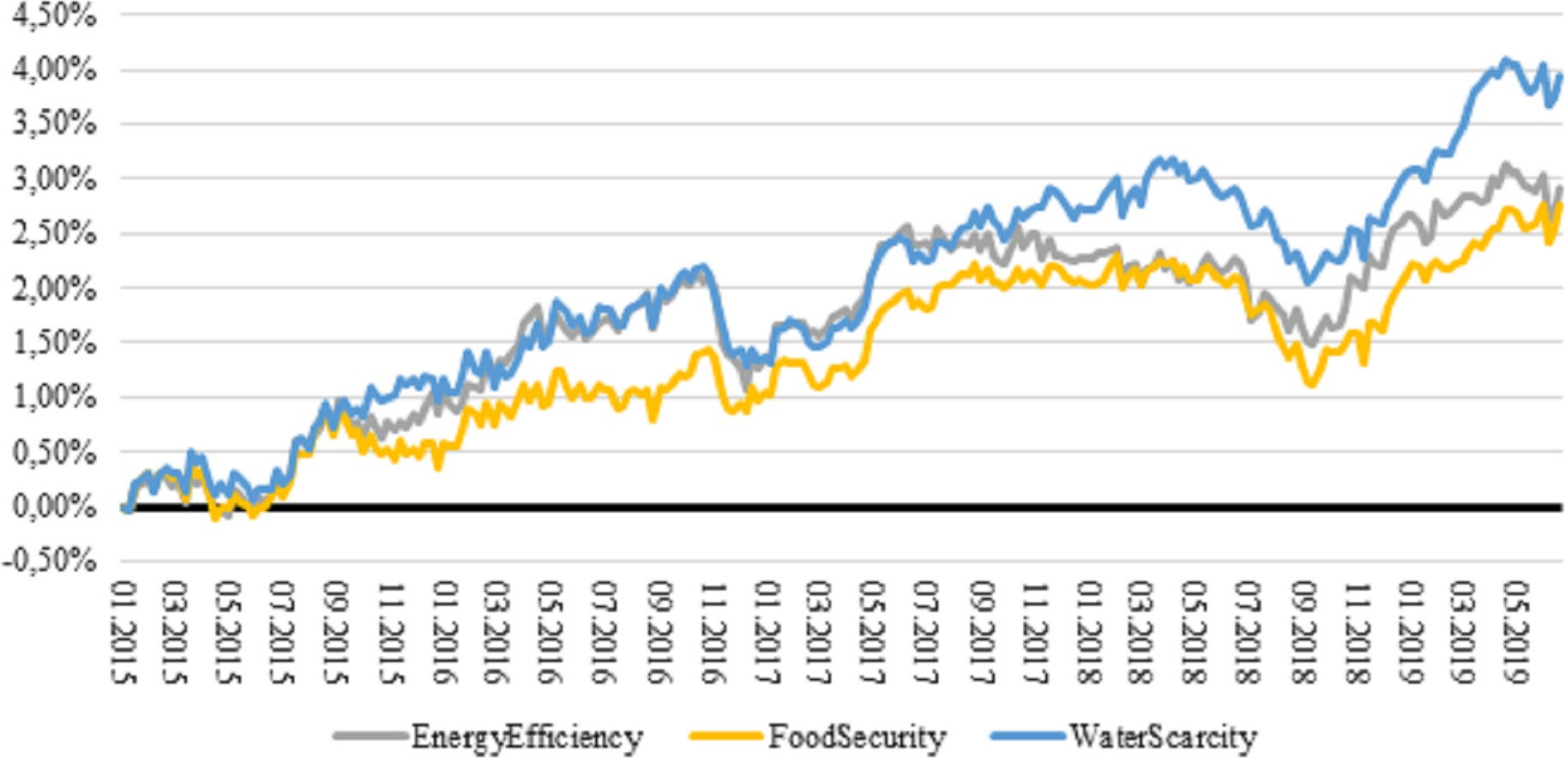
Cumulative market-relative return of environment megatrends, 2015–2019. Returns are total log returns. MXWD is the Bloomberg ticker for MSCI ACWI Index.

Before presenting our results, we visualise the performance of megatrend factors compared to the benchmark (MSCI ACWI Index). Fig 2 depicts the cumulative market-relative total log return of the three environmental megatrends introduced previously. We emphasise that these returns are pure, that is, exposure values to style, industry and country factors are the same as the values of the benchmark. One can see that each E megatrend realised positive market-relative returns, among which water scarcity yielded the highest return (3.94 per cent). Energy efficiency was the second-best strategy with a cumulative return of 2.91 per cent. Food security megatrend portfolio ranked third. However, it also outperformed the benchmark by 2.76 per cent. Nevertheless, the megatrends tended to move together, i.e. there is a high and positive correlation between the returns.

The general belief among market participants is that ESG-themed investments impact our lives in the long-term, as megatrends are structural shifts; therefore, the possible higher performance should also prevail in the long run. The chart shows, however, that we already live ‘in the long run’, meaning that companies offering solutions on environmental challenges perform relatively well.

**Fig 3:**
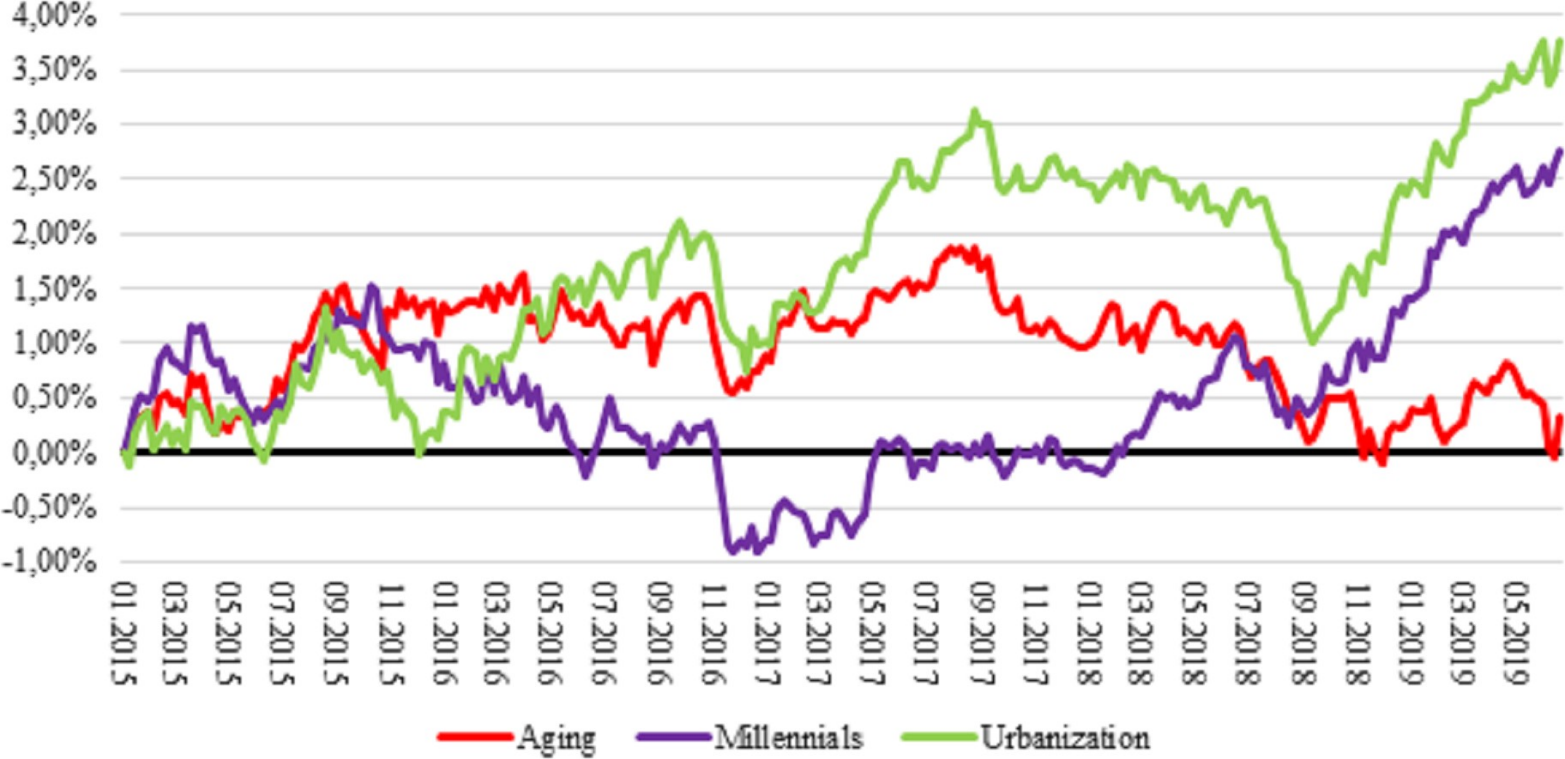
Cumulative market-relative return of social megatrends, 2015–2019. Returns are total log returns. MXWD is the Bloomberg ticker for MSCI ACWI Index.

Fig 3 illustrates the performance of social megatrends. Urbanisation and millennials megatrends achieved a return of 3.76 and 2.76 per cent, respectively. Urbanisation megatrend did well during the past 4.5 years, but millennials was an underperformer from June 2016 till March 2018. Ageing had a stable 1.00–1.50 per cent surplus over the benchmark, but during 2019 this extra return vanished.

**Fig 4:**
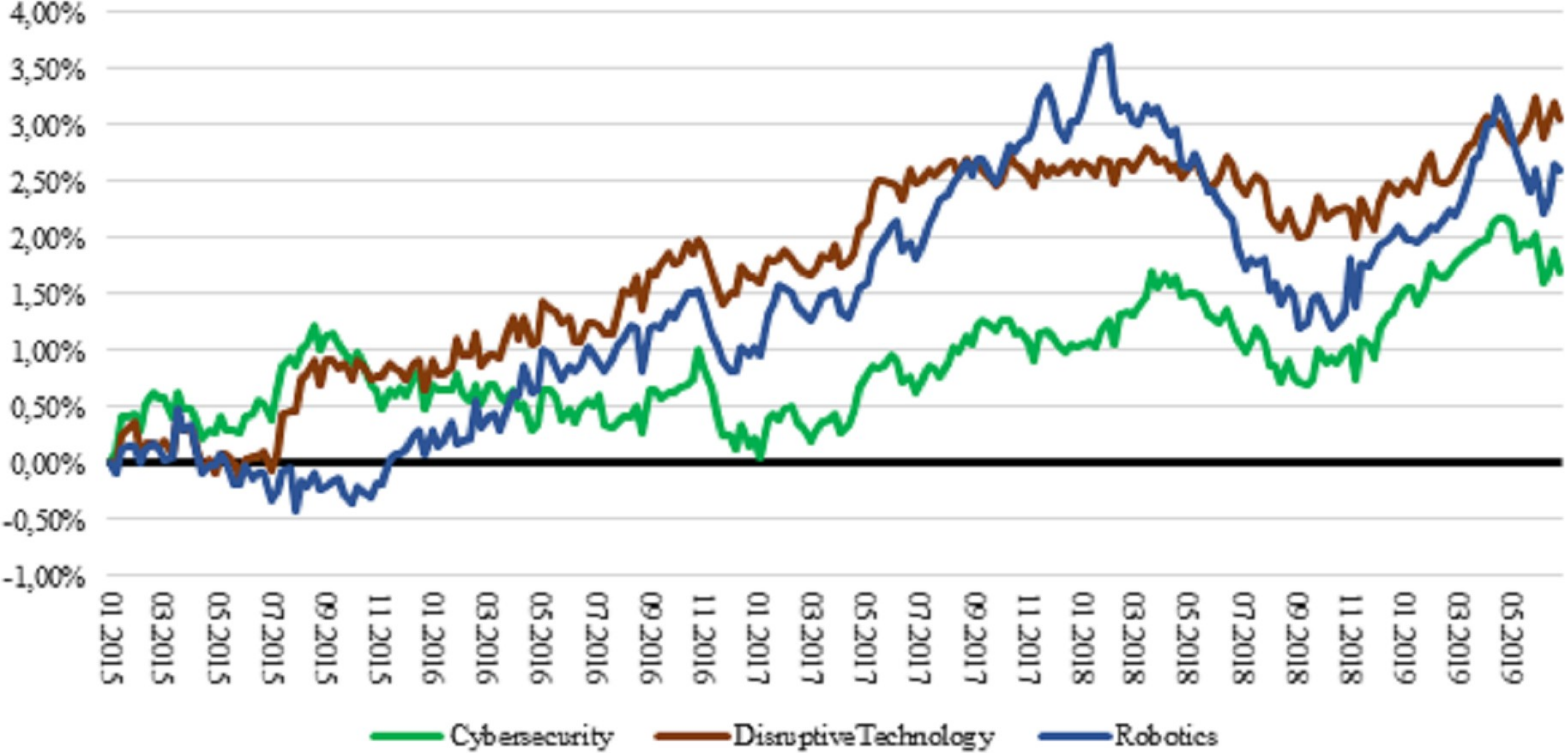
Cumulative market-relative return of governance megatrends, 2015–2019. Returns are total log returns. MXWD is the Bloomberg ticker for MSCI ACWI Index.

Governance megatrends also outperformed the market (Fig 4), though robotics megatrend was more volatile than cybersecurity and disruptive technology. Disruptive technology yielded 3.05 per cent excess return above the market, which was the highest among governance megatrends (robotics: 2.60 per cent; cybersecurity: 1.70 per cent).

Tables 4 and 5 summarises the regression results of OLS (with HAC standard errors) and GMM-IV_d_ for (18)-(21). Comparing the ESG-themed factor portfolio returns to the passive strategy (Panel A and E), each environmental megatrend (energy efficiency, food security, water scarcity) and the disruptive technologies megatrend outperformed the market significantly. The performance of energy efficiency and food security megatrend is statistically significant at 10.00 per cent; while water scarcity and disruptive technology are significant at 5.00 per cent. None of the social megatrend alphas is significant; further, cybersecurity and robotics do not have significant figures either. Measuring the Sharpe ratios, two megatrends, water scarcity and disruptive technologies remained significant. The t-statistics, however, decreased: the disruptive technologies megatrend is significant only at 10.00 per cent; the energy efficiency and food security themes are not significant any more at the usual significance levels.

**Table 4:** Regressions of megatrend portfolios on pure factor portfolios via OLS.

Factors	EE	FS	WS	AG	MI	UR	CY	DT	RO
***A*: *CAPM***									
Alpha	0.602*	0.590*	0.841**	0.177	0.547	0.851	0.404	0.666**	0.495
* *	*1*.*76*	*1*.*89*	*2*.*24*	*0*.*47*	*1*.*11*	*1*.*58*	*1*.*35*	*2*.*40*	*0*.*92*
MRP	1.000***	1.000***	1.000***	1.000***	1.000***	0.990***	1.000***	1.000***	1.010***
* *	163.41	163.54	147.69	130.76	219.07	121.00	200.39	186.92	170.31
**Sharpe**	0.565	0.559	0.580**	0.522	0.557	0.576	0.547	0.565*	0.548
* *	*1*.*63*	*1*.*57*	*2*.*07*	*0*.*20*	*1*.*04*	*1*.*48*	*1*.*04*	*1*.*89*	*0*.*88*
***B*: *Fama-French-Carhart (FFC)***									
Alpha	0.270	0.219	0.471	-0.217	0.175	0.438	0.036	0.274	0.124
* *	*0*.*67*	*0*.*68*	*1*.*18*	*-0*.*67*	*0*.*32*	*0*.*77*	*0*.*13*	*1*.*01*	*0*.*24*
MRP	1.000***	1.000***	1.000***	1.000***	1.010***	1.000***	1.000***	1.000***	1.010***
* *	*167*.*61*	*178*.*73*	*147*.*03*	*155*.*58*	*222*.*28*	*114*.*04*	*240*.*43*	*236*.*24*	*231*.*43*
SIZE	0.031	0.045***	0.050***	0.049**	0.037	0.083**	0.039	0.046***	0.041*
* *	*1*.*01*	*2*.*90*	*2*.*91*	*2*.*37*	*1*.*50*	*2*.*27*	*1*.*46*	*3*.*31*	*1*.*72*
VALUE	0.186***	0.221***	0.189***	0.223***	0.191***	0.188***	0.207***	0.237******	0.218***
* *	*5*.*66*	*7*.*10*	*5*.*00*	*6*.*34*	*4*.*85*	*3*.*53*	*7*.*28*	*8*.*15*	*8*.*15*
MOM	0.109***	0.107***	0.117***	0.118***	0.129***	0.12***	0.116***	0.112***	0.111***
* *	*4*.*53*	*5*.*40*	*5*.*23*	*5*.*77*	*3*.*71*	*4*.*40*	*7*.*16*	*8*.*11*	*4*.*75*
***C*: *Fama-French 5-factor model***									
Alpha	0.006	-0.027	0.223	-0.439	-0.084	0.109	-0.137	0.089	-0.115
* *	*0*.*02*	*-0*.*15*	*0*.*81*	*-1*.*56*	*-0*.*17*	*0*.*30*	*-0*.*67*	*0*.*45*	*-0*.*29*
MRP	0.990***	0.990***	1.000***	0.990***	1.000***	0.990***	0.990***	0.990***	1.010***
* *	*155*.*86*	*189*.*64*	*153*.*13*	*169*.*91*	*196*.*48*	*117*.*14*	*251*.*40*	*254*.*44*	*261*.*46*
SIZE	0.008	0.026	0.029	0.036	0.017	0.043	0.021	0.024*	0.021
* *	*0*.*30*	*1*.*59*	*1*.*60*	*1*.*38*	*0*.*59*	*1*.*53*	*0*.*67*	*1*.*68*	*0*.*95*
VALUE	0.055**	0.097***	0.059**	0.102**	0.052*	0.028	0.095***	0.123***	0.093**
* *	*2*.*41*	*4*.*23*	*2*.*04*	*2*.*48*	*1*.*74*	*0*.*70*	*4*.*90*	*4*.*33*	*2*.*14*
PROFIT	0.171***	0.163***	0.163***	0.156***	0.175***	0.193***	0.116***	0.115***	0.157***
* *	*5*.*84*	*4*.*74*	*4*.*30*	*4*.*42*	*3*.*75*	*3*.*73*	*3*.*37*	*3*.*83*	*2*.*90*
INV	0.350***	0.325***	0.360***	0.319***	0.378***	0.466***	0.339***	0.354***	0.342***
* *	*7*.*55*	*10*.*73*	*10*.*75*	*7*.*82*	*10*.*10*	*11*.*67*	*10*.*40*	*10*.*96*	*8*.*15*
***D*: *Fama-French 5-factor model*, *augmented with a liquidity factor***									
Alpha	0.000	-0.041	0.189	-0.478	-0.112	0.063	-0.119	0.075	-0.13
* *	*0*.*00*	*-0*.*24*	*0*.*69*	*-1*.*60*	*-0*.*23*	*0*.*17*	*-0*.*55*	*0*.*39*	*-0*.*34*
MRP	0.990***	0.990***	1.000***	0.990***	1.000***	0.990***	0.990***	0.990***	1.010***
* *	*154*.*57*	*193*.*72*	*154*.*86*	*168*.*89*	*198*.*39*	*116*.*81*	*256*.*04*	*254*.*30*	*275*.*43*
SIZE	0.005	0.019	0.012	0.016	0.003	0.019	0.030	0.016	0.013
* *	*0*.*16*	*0*.*92*	*0*.*54*	*0*.*48*	*0*.*09*	*0*.*54*	*0*.*96*	*0*.*81*	*0*.*34*
VALUE	0.055**	0.097***	0.058*	0.102**	0.051*	0.0270	0.096***	0.123***	0.093**
* *	*2*.*38*	*4*.*11*	*1*.*93*	*2*.*44*	*1*.*70*	*0*.*66*	*5*.*05*	*4*.*31*	*2*.*11*
PROFIT	0.170***	0.163***	0.162***	0.155***	0.174***	0.192***	0.116***	0.115***	0.157***
* *	*5*.*84*	*4*.*70*	*4*.*12*	*4*.*24*	*3*.*77*	*3*.*66*	*3*.*47*	*3*.*75*	*2*.*92*
INV	0.350***	0.324***	0.358***	0.316***	0.376***	0.463***	0.340***	0.353***	0.341***
* *	*7*.*58*	*10*.*33*	*10*.*18*	*7*.*27*	*9*.*66*	*11*.*99*	*10*.*67*	*10*.*52*	*7*.*52*
LIQ	0.004	0.009	0.020	0.024	0.017	0.028	-0.011	0.008	0.009
* *	*0*.*23*	*0*.*61*	*1*.*25*	*0*.*86*	*0*.*63*	*1*.*28*	*-0*.*76*	*0*.*53*	*0*.*26*

Both alphas (log returns) and Sharpe-ratios are annualised figures. Alphas are expressed in percentage points (e.g. an alpha of 0.15 is 15 basis points per year).

EE–Energy efficiency, FS–Food security; WS–Water scarcity; AG–Ageing; MI–Millennials; UR–Urbanisation; CY–Cybersecurity; DT- Disruptive technology; RO–Robotics.

Standard errors (SE) are Newey-West (HAC) standard errors. The coefficient t-statistics are in italics.

*** p < 0.01

** p < 0.05

* p < 0.10.

**Table 5:** Regressions of megatrend portfolios on pure factor portfolios via GMM-IV_d_.

Factors	EE	FS	WS	AG	MI	UR	CY	DT	RO
***E*: *CAPM***									
Alpha	0.600*	0.590*	0.840**	0.190	0.540	0.840	0.400	0.670**	0.490
* *	*1*.*71*	*1*.*85*	*2*.*15*	*0*.*52*	*1*.*08*	*1*.*52*	*1*.*28*	*2*.*37*	*1*.*04*
MRP	1.000***	1.000***	1.000***	1.000***	1.010***	1.000***	1.000***	1.000***	1.010***
	163.40	161.91	159.91	126.97	180.77	111.03	181.07	174.25	151.11
***F*: *Fama-French-Carhart (FFC)***									
Alpha	0.263	0.214	0.455	-0.209	0.158	0.425	0.024	0.271	0.119
* *	*0*.*68*	*0*.*69*	*1*.*15*	*-0*.*61*	*0*.*30*	*0*.*79*	*0*.*09*	*1*.*03*	*0*.*27*
MRP	1.000***	1.000***	1.000***	1.000***	1.010***	1.000***	0.990***	1.000***	1.010***
* *	*174*.*24*	*182*.*94*	*172*.*03*	*139*.*11*	*155*.*91*	*107*.*27*	*178*.*60*	*201*.*95*	*161*.*96*
SIZE	0.030	0.048***	0.060***	0.052*	0.048**	0.083**	0.054**	0.050***	0.051*
* *	*0*.*98*	*2*.*62*	*2*.*77*	*1*.*89*	*2*.*05*	*2*.*27*	*2*.*25*	*3*.*27*	*1*.*68*
VALUE	0.182***	0.217***	0.182***	0.22***	0.185***	0.178***	0.203***	0.237***	0.214***
* *	*6*.*16*	*7*.*44*	*5*.*34*	*6*.*53*	*4*.*58*	*3*.*68*	*6*.*92*	*8*.*12*	*6*.*52*
MOM	0.112***	0.113***	0.129***	0.117***	0.139***	0.123***	0.124***	0.121***	0.120***
* *	*5*.*94*	*7*.*81*	*7*.*93*	*5*.*84*	*5*.*85*	*6*.*23*	*9*.*59*	*10*.*55*	*4*.*99*
***G*: *Fama-French 5-factor model***									
Alpha	0.047	0.011	0.247	-0.381	-0.059	0.145	-0.114	0.124	-0.099
* *	*0*.*16*	*0*.*06*	*0*.*88*	*-0*.*96*	*-0*.*13*	*0*.*37*	*-0*.*45*	*0*.*62*	*-0*.*24*
MRP	0.990***	0.990***	1.000***	0.990***	1.000***	0.990***	0.990***	0.990***	1.000***
* *	*161*.*60*	*195*.*17*	*175*.*30*	*168*.*72*	*174*.*22*	*107*.*48*	*178*.*32*	*209*.*67*	*171*.*46*
SIZE	0.012	0.033*	0.040**	0.041	0.025	0.055	0.036	0.031**	0.029
* *	*0*.*40*	*1*.*84*	*2*.*00*	*1*.*51*	*0*.*83*	*1*.*62*	*1*.*40*	*2*.*11*	*1*.*19*
VALUE	0.057**	0.107***	0.063**	0.123***	0.055	0.024	0.102***	0.130***	0.117**
* *	*2*.*26*	*4*.*30*	*2*.*07*	*2*.*93*	*1*.*48*	*0*.*65*	*3*.*31*	*4*.*06*	*2*.*55*
PROFIT	0.156***	0.149***	0.155***	0.135***	0.173***	0.173***	0.107***	0.107***	0.145***
* *	*4*.*51*	*4*.*29*	*3*.*91*	*3*.*13*	*3*.*25*	*3*.*84*	*2*.*91*	*3*.*26*	*2*.*83*
INV	0.331***	0.309***	0.347***	0.294***	0.363***	0.453***	0.334***	0.345***	0.344***
* *	*6*.*67*	*9*.*13*	*9*.*76*	*6*.*66*	*9*.*66*	*10*.*34*	*8*.*54*	*10*.*32*	*6*.*70*
***H*: *Fama-French 5-factor model*, *augmented with a liquidity factor***									
Alpha	0.04	-0.008	0.197	-0.437	-0.108	0.077	-0.117	0.1	-0.123
* *	*0*.*13*	*-0*.*04*	*0*.*69*	*-1*.*16*	*-0*.*24*	*0*.*20*	*-0*.*47*	*0*.*52*	*-0*.*30*
MRP	1.000***	0.990***	1.000***	0.990***	1.000***	0.990***	0.990***	0.990***	1.000***
* *	*166*.*34*	*208*.*91*	*187*.*94*	*162*.*56*	*179*.*69*	*115*.*52*	*177*.*57*	*217*.*97*	*182*.*44*
SIZE	0.010	0.025	0.021	0.026	0.005	0.034	0.038	0.024	0.016
* *	*0*.*31*	*1*.*16*	*0*.*97*	*0*.*79*	*0*.*17*	*0*.*92*	*1*.*39*	*1*.*13*	*0*.*42*
VALUE	0.059**	0.107***	0.062*	0.122***	0.055	0.024	0.102***	0.129***	0.116***
* *	*2*.*37*	*4*.*22*	*1*.*96*	*2*.*96*	*1*.*46*	*0*.*61*	*3*.*55*	*4*.*02*	*2*.*59*
PROFIT	0.152***	0.146***	0.151***	0.131***	0.169***	0.168***	0.106***	0.106***	0.145***
* *	*4*.*38*	*4*.*18*	*3*.*59*	*2*.*93*	*3*.*22*	*3*.*46*	*2*.*91*	*3*.*10*	*2*.*95*
INV	0.325***	0.306***	0.342***	0.286***	0.358***	0.444***	0.332***	0.343***	0.344***
* *	*6*.*64*	*8*.*94*	*9*.*34*	*6*.*24*	*8*.*87*	*10*.*34*	*8*.*70*	*10*.*09*	*6*.*03*
LIQ	0.003	0.011	0.027	0.027	0.027	0.034	0	0.012	0.015
* *	*0*.*19*	*0*.*74*	*1*.*60*	*0*.*99*	*1*.*07*	*1*.*50*	*-0*.*01*	*0*.*74*	*0*.*39*

Notes: Alphas (log returns) are annualised figures and expressed in percentage points (e.g. an alpha of 0.15 is 15 basis points per year).

EE–Energy efficiency, FS–Food security; WS–Water scarcity; AG–Ageing; MI–Millennials; UR–Urbanisation; CY–Cybersecurity; DT- Disruptive technology; RO–Robotics.

Standard errors (SE) are Newey-West (HAC) standard errors. The coefficient t-statistics are in italics.

*** p < 0.01

** p < 0.05

* p < 0.10.

Looking at the Fama-French-Carhart 4-factor model (Panel B and F), the alphas are still positive for each megatrend, except for ageing. Ageing has a negative alpha of 21.7 basis points p.a. in OLS and 20.9 basis points p.a. in GMM-IV_d_ setting. Nevertheless, none of the alphas is significantly different from zero; thus, our ESG-themed factor portfolios achieved at least comparable risk-adjusted returns to what FFC 4-factor model suggests.

The figures of the Fama-French 5-factor model (Panel C and G) are more heterogeneous than the previous ones, as five megatrends (food security, ageing, millennials, cybersecurity, and robotics) underperformed the market in the OLS and four (ageing, millennials, cybersecurity, and robotics) realised negative alphas in the GMM-IV_d_ context. Based on GMM-IV_d_, each environmental portfolio still has positive alphas. Once again, these alphas are insignificant at the usual statistical significance levels.

Finally, we introduce the results of the liquidity factor augmented FF5 model. The conclusions are almost the same as the ‘plain’ FF5 model: the same five megatrends yielded negative returns via OLS, but now the GMM-IV_d_ estimation results in negative alpha for food security as well. However, none of the alphas is significant statistically.

Turning to the explanatory factors’ coefficients, one can see that the betas are equal to one in almost every case (in fact, they do not statistically differ from one), which is the consequence of our PFP construction technique: we control for beta risk (see Table 2), meaning that megatrend portfolios are beta neutral, viz., they have the same beta as the market (i.e. 1).

The momentum factor is significant at 1.00 per cent level both with the OLS and GMM-IV_d_ estimation method. The coefficients of the size factor in the FFC model calculated via OLS and GMM-IV_d_ are significant in the case of six and eight megatrends, respectively. In average, the coefficients are a bit higher for GMM-IV_d_. The impact of the size coefficient in the FF5 model is almost entirely insignificant using OLS, and insignificant for six megatrends with GMM-IV_d_ (again, coefficient values are somewhat higher in GMM context). The FF5 model augmented with a liquidity measure suggests that size becomes insignificant regardless of using the OLS or GMM-IV_d_ estimator. The value factor in the FFC model is significant for each megatrend at 1 per cent with both estimator; however, they are now higher for OLS. In the FF5 and FF5L model, the value factor is significant for eight megatrends of OLS and six megatrends of GMM-IV_d_. The profitability and investment factors are very significant (p<0.01) either calculated by OLS or GMM-IV_d_. The liquidity factor of OLS and GMM-IV_d_ is not significant for any megatrends, which is mostly in line with [32, 99].

Based on our GMM-IV_d_ estimates, the coefficients for value, investment and profitability are significant which are in contrary to *Racicot et al*. [34, 98, 99] who found that in most cases the market factor is the only variable which has significant explanatory power. The authors highlight that measurement errors may be the reason for their results. To test errors-in-variables (EIV), they suggest using a Hausman_d_ procedure. We executed the calculations and found that the residuals’ (ω’s) t statistics are mostly not significant in our analysis, which indicates that there are, at most, modest measurement errors. However, we also calculated F tests to see if collectively, none of the ω coefficients in the artificial regressions is significantly different from zero. We found that the F statistics indicate measurement errors in the case of five megatrends, including food security, ageing, and each technological governance megatrend. (The Hausman artificial regression tests can be found in S6 Appendix in S2 File along with relevance and exogeneity tests of the IVs).

Besides measurement errors, the GMM-IV_d_ method is also a useful analysis tool in our study, since none of the variables are normally distributed. (We calculated the Jarque-Bera statistic for both the dependent and explanatory variables and found that the values of the statistic are greater than 5.99, which is the critical value of the Chi-Square distribution at 5 per cent level for 2 degrees of freedom). *Racicot and Rentz* [34, p. 58] emphasise that non-normality supports the logic of the method, which uses higher moments (cumulants) as instruments for the GMM estimation process.

Table 6 contains the summary of the ESG-themed megatrend factor portfolios’ alpha estimates. The takeaway message is that 50 model specification out of 72 resulted in positive alphas, although only four CAPM alpha is statistically significant. Three ESG-themed megatrends (water scarcity, urbanisation, disruptive technology) have a positive alpha value regardless of model specification. Note that the alphas are less sensitive to the estimation method applied, which is in line with [32].

**Table 6:** Summary of alphas via OLS and GMM-IV_d_.

Model	EE	FS	WS	AG	MI	UR	CY	DT	RO
***OLS***									
CAPM	0.602*	0.590*	0.841**	0.178	0.548	0.851	0.404	0.666**	0.495
FFC	0.271	0.219	0.472	-0.217	0.176	0.438	0.037	0.274	0.124
FF5	0.007	-0.027	0.223	-0.439	-0.084	0.109	-0.138	0.090	-0.116
FF5L	-0.000	-0.042	0.189	-0.478	-0.112	0.063	-0.119	0.076	-0.131
***GMM-IV***_***d***_									
CAPM	0.600*	0.589*	0.839**	0.190	0.540	0.840	0.400	0.670**	0.490
FFC	0.264	0.214	0.455	-0.210	0.159	0.425	0.025	0.272	0.120
FF5	0.048	0.012	0.248	-0.382	-0.060	0.146	-0.115	0.124	-0.100
FF5L	0.040	-0.008	0.198	-0.438	-0.109	0.077	-0.117	0.101	-0.124

Notes: Alphas (log returns) are annualised figures and expressed in percentage points (e.g. an alpha of 0.15 is 15 basis points per year).

The green and red figures represent positive and negative alphas, respectively.

*** p < 0.01

** p < 0.05

* p < 0.10.

The impact of transaction costs is an essential consideration in assessing the profitability of trading strategies [121]; therefore, we analysed the ESG-themed megatrend portfolios after controlling for costs and fees. We concentrate on the expense ratio, as did two recent studies [122, 123]. We follow the method of *Derwall et al*. and *Kemp-Osthoff* [124, 125] assuming an expense ratio between 25 and 150 basis points which are slightly lower than in the studies (50 and 200 basis points). In fact, these expense ratios are in line with what *Alda* [122] and *Brakman Reiser-Tucker* [123] found typical nowadays for ESG ETFs. The expense-adjusted portfolio return is the ESG-themed megatrend return minus the sum of transaction costs.

Table 7 provides the performance statistics in the same manner as Table 6. The alphas decrease as the transaction cost increase. If we assume an annual 25 basis points expense ratio, the ageing megatrend has a statistically significant negative alpha for FF5 and FF5L models (GMM-IV_d_); nevertheless, the other megatrend alphas do not significantly differ from zero. Calculating with a 50 basis points expense rate the food security, ageing and cybersecurity megatrend yield significant negative alphas (again, in FF5 and FF5L and by GMM-IV_d_). The 1.00 and 1.50 percentage points scenarios show significant underperformance in almost every model specification. According to *Morningstar* [126], in practice, the least expensive ESG-themed ESG funds have an expense ratio of around 0.50–0.60 percentage points, while the median is approximately 1.00 percentage. Assuming the ‘low-cost’ case, we see that six megatrend alphas are not statistically different from zero and only three underperform significantly.

**Table 7:** Summary of alphas via OLS and GMM-IV_d_ after controlling for transaction costs.

Model	EE	FS	WS	AG	MI	UR	CY	DT	RO
***25 basis points***									
***OLS***									
CAPM	0.352	0.340	0.591	-0.072	0.297	0.601	0.154	0.416	0.245
FFC	0.020	-0.030	0.221	-0.467	-0.074	0.188	-0.213	0.024	-0.125
FF5	-0.243	-0.277	-0.026	-0.689**	-0.334	-0.140	-0.387*	-0.160	-0.365
FF5L	-0.249	-0.2919*	-0.061	-0.7283**	-0.362	-0.187	-0.3692*	-0.174	-0.381
***GMM-IV***									
CAPM	0.350	0.341	0.592	-0.058	0.291	0.590	0.158	0.423	0.246
FFC	0.013	-0.035	0.205	-0.459	-0.091	0.175	-0.225	0.021	-0.130
FF5	-0.202	-0.238	-0.002	-0.631*	-0.309	-0.104	-0.364	-0.125	-0.349
FF5L	-0.209	-0.258	-0.052	-0.687*	-0.358	-0.172	-0.367	-0.149	-0.373
**Model**	**EE**	**FS**	**WS**	**AG**	**MI**	**UR**	**CY**	**DT**	**RO**
***50 basis points***									
**OLS**									
CAPM	0.102	0.090	0.341	-0.322	0.047	0.351	-0.095	0.166	-0.004
FFC	-0.229	-0.280	-0.028	-0.717**	-0.324	-0.061	-0.463*	-0.225	-0.375
FF5	-0.493	-0.527***	-0.276	-0.939***	-0.584	-0.390	-0.637***	-0.410**	-0.615
FF5L	-0.499	-0.5419***	-0.311	-0.9783***	-0.612	-0.437	-0.6192***	-0.4241**	-0.631
**GMM-IV**									
CAPM	0.100	0.091	0.342	-0.308	0.041	0.340	-0.091	0.173	-0.003
FFC	-0.236	-0.285	-0.044	-0.709*	-0.341	-0.074	-0.475	-0.228	-0.380
FF5	-0.452	-0.488*	-0.252	-0.881**	-0.559	-0.354	-0.614**	-0.375	-0.599
FF5L	-0.459	-0.508**	-0.302	-0.937**	-0.608	-0.422	-0.617**	-0.399	-0.623
***100 basis points***									
**OLS**									
CAPM	-0.397	-0.409	-0.158	-0.822**	-0.452	-0.148	-0.595**	-0.333	-0.504
FFC	-0.729*	-0.78**	-0.528	-1.217***	-0.824	-0.561	-0.963***	-0.725***	-0.875*
FF5	-0.993***	-1.027***	-0.776***	-1.439***	-1.084**	-0.890**	-1.137***	-0.910***	-1.115***
FF5L	-0.999***	-1.0419***	-0.8105***	-1.4783***	-1.1123**	-0.9369**	-1.1192***	-0.9241***	-1.1309***
**GMM-IV**									
CAPM	-0.399	-0.408	-0.157	-0.808*	-0.458	-0.159	-0.591	-0.326	-0.503
FFC	-0.736**	-0.785***	-0.544	-1.209***	-0.841*	-0.574	-0.975***	-0.728***	-0.88**
FF5	-0.952***	-0.988***	-0.752**	-1.381***	-1.059**	-0.854**	-1.114***	-0.875***	-1.099***
FF5L	-0.959***	-1.008***	-0.802***	-1.437***	-1.108***	-0.922**	-1.117***	-0.899***	-1.123***
***150 basis points***									
**OLS**									
CAPM	-0.897***	-0.909***	-0.658*	-1.322***	-0.952*	-0.648	-1.095***	-0.833***	-1.004*
FFC	-1.229***	-1.28***	-1.028**	-1.717***	-1.324**	-1.061*	-1.463***	-1.225***	-1.375***
FF5	-1.493***	-1.527***	-1.276***	-1.939***	-1.584***	-1.39***	-1.637***	-1.41***	-1.615***
FF5L	-1.499***	-1.541***	-1.31***	-1.978***	-1.612***	-1.436***	-1.619***	-1.424***	-1.63***
**GMM-IV**									
CAPM	-0.899**	-0.908***	-0.657*	-1.308***	-0.958*	-0.659	-1.091***	-0.826**	-1.003**
FFC	-1.236***	-1.285***	-1.044***	-1.709***	-1.341***	-1.074**	-1.475***	-1.228***	-1.38***
FF5	-1.452***	-1.488***	-1.252***	-1.881***	-1.559***	-1.354***	-1.614***	-1.375***	-1.599***
FF5L	-1.459***	-1.508***	-1.302***	-1.937***	-1.608***	-1.422***	-1.617***	-1.399***	-1.623***

Notes: Alphas (log returns) are annualised figures and expressed in percentage points (e.g. an alpha of 0.15 is 15 basis points per year).

The green and red figures represent positive and negative alphas, respectively.

*** p < 0.01

** p < 0.05

* p < 0.10.

In summary, our findings show that most megatrends yielded at least comparable returns to the benchmark after accounting for risk but before accounting for transactions costs. We can say that there is at least neutral relationship between ESG and market performance, which supports the hypothesis that ESG risks can be diversified, or ESG companies have a low level of idiosyncratic risk (see *Boutin-Dufresne and Savaria* [127]). Further, these findings support the hypothesis of *Diltz* that there is no under-diversification effect due to the immense size and ample liquidity of the equity markets [128]. Alternatively, one could conclude that the EMH holds.

Further, the results also suggest that investors should recognise ESG investing as a superior strategy relative to conventional approaches as they can attain comparable financial performance and still address ESG concerns. The results are in line with the findings of *Revelli-Viviani* [91], *Martí-Ballester* [5]. Thematic investing can help in achieving UN’s Sustainable Development Goals (SDGs) such as ‘end hunger, achieve food security’ (SDG2), ‘ensure healthy lives and promote well-being for all at all ages’ (SDG3), ‘make cities and human settlements inclusive, safe, resilient and sustainable’ (SDG11) [3]. Besides our findings, it is a common opinion among practitioners that megatrends ought to work out in the long run [129]. Our results are not in contradiction to this notion. Also, we pinpoint that megatrend factors by themselves are not a recipe for outperformance, at least after adjusting for transaction costs.

Environmental megatrends and disruptive technologies outperformed the passive strategy. This finding is against the semi-strong form of EMH and supports the ‘doing well while doing good’ concept of *Hamilton et al*. [62]. We recommend, in line with *Renneboog*, *Horst and Zhang* [52], that investors ought to pursue fundamental research to determine the asset allocation among the winning megatrends to enhance investor returns further. Besides, due to transaction costs, the surplus can quickly vanish, meaning that they should carefully analyse the market to discover cheap opportunities.

The positive alphas and Sharpe-ratios show that investors are turning to thematic investments in a hunt for stocks with a particular quality attribute that supersedes the advantages of the traditional style investments: seeking investments that are well-positioned to benefit from secular growth, that can surpass economic cycles. However, we are yet to see how these megatrend factors fare in a real market decline. Nevertheless, according to the business press and some new academic studies, during the first wave of COVID-19 ESG-themed portfolios were resilient and outperformed the market [130–132].

## 7. Conclusion

We emphasise the growing importance of ESG themed megatrend investments. Megatrends are secular, transformative processes that have the potential to impact the environment, the economy, and society at large. To verify the validity of megatrend investing, we define nine themes with the three E, S, and G related megatrends. These are as follows: energy efficiency, food security, water scarcity (environmental megatrends); ageing, millennials, urbanisation (social megatrends); cybersecurity, disruptive technologies, robotics (governance megatrends).

We introduce a quantification of stock megatrend exposures (MTE) drawing on signalling theory. Based on our analysis, portfolio managers’ (i.e. sellers) stock selection practices indicate (signal) to investors and analysts (i.e. buyers) that the companies they have selected are a suitable proxy for megatrend investment. Consequently, the relative amount of money inflows into megatrend funds signals the market’s belief that those stocks are the best candidates to represent megatrends.

The research question examines whether ESG themed megatrend investing can be a tool to align sustainability goals of investors based on the UN Sustainable Development Goals (SDGs) without sacrificing returns. To this end, we test whether megatrend factor portfolios could generate superior returns, on a risk-adjusted basis and accounting for transaction costs. We first compare the returns to the passive strategy (viz., we calculate CAPM alphas and Sharpe ratios relative to the market benchmark), and then measure the alpha applying various Fama-French model specifications (e.g. FF three-factor model, FF five-factor model). Our research question can also be interpreted as a test of the efficient market hypothesis (EMH).

Filtering out secondary factor exposures and isolating the effects of ESG factors is a crucial methodological requirement; therefore, we use the pure factor portfolio methodology that applies multivariate cross-sectional regression equations following the Fama-MacBeth procedure. Pure factor portfolios are fully invested long-short factor mimicking portfolios. One of the critical methodological challenges is how to estimate the coefficients in times series analysis. We applied two methods: traditional OLS with Newey-West (HAC) standard errors, and generalised method of moments using innovative, robust distance instrumental variables (GMM-IV_d_). The GMM-IV_d_ method is suitable to address the various manifestations of endogeneity inherent in factor models. According to the literature, the GMM-IV_d_ approach provides solutions to measurement errors (errors-in-variables) and other types of endogeneity. Further, it also handles non-normality as it uses higher moments (cumulants) as instrumental variables for the GMM estimation process.

One important result is that most of the megatrend factors yielded non-negative excess returns compared to the MSCI All Country Index benchmark, even after accounting for transaction costs up to 50bps/annum. The implication of this result is that ESG risks can be diversified and no extra costs are borne for sustainability aligned investors. Some of these sustainability goals include ‘end hunger, achieve food security’ (SDG2), ‘ensure healthy lives and promote well-being for all at all ages’ (SDG3), ‘make cities and human settlements inclusive, safe, resilient and sustainable’ (SDG11).

Higher transaction costs, as is the case for some of these ETFs with expense ratios reaching 80-100bps, may be an indication of two things: ESG themed megatrend investors were willing to sacrifice ca. 30-50bps of annual return to remain aligned with sustainability targets, or that expense ratio may well decline in the future.

We find no evidence that ESG alignment of companies adds material idiosyncratic risk. Further, these findings support the literature that there is no under-diversification effect due to the massive size and ample liquidity of markets. This is in line with at least the weak form of EMH.

## Supporting information

S1 File Supporting data. (XLSX)

S2 File (DOCX)
