# Supplementary material for: Performance measurement of ESG-themed megatrend investments in global equity markets using pure factor portfolios methodology
Source: PLoS One. 2020 Dec 22;15(12):e0244225. doi: 10.1371/journal.pone.0244225 (PMC7755284; doi:10.1371/journal.pone.0244225)
Supplement: S2 File — (DOCX) [file pone.0244225.s002.docx]

**S1 Appendix. United Nations’ Sustainable Development Goals (SDGs) form the Agenda ‘Transforming our world: the 2030 Agenda for Sustainable Development’**

| Goal 1. | End poverty in all its forms everywhere |
| --- | --- |
| Goal 2. | End hunger, achieve food security and improved nutrition and promote sustainable agriculture |
| Goal 3. | Ensure healthy lives and promote well-being for all at all ages |
| Goal 4. | Ensure inclusive and equitable quality education and promote lifelong learning opportunities for all |
| Goal 5. | Achieve gender equality and empower all women and girls |
| Goal 6. | Ensure availability and sustainable management of water and sanitation for all |
| Goal 7. | Ensure access to affordable, reliable, sustainable and modern energy for all |
| Goal 8. | Promote sustained, inclusive and sustainable economic growth, full and productive employment and decent work for all |
| Goal 9. | Build resilient infrastructure, promote inclusive and sustainable industrialisation and foster innovation |
| Goal 10. | Reduce inequality within and among countries |
| Goal 11. | Make cities and human settlements inclusive, safe, resilient and sustainable |
| Goal 12. | Ensure sustainable consumption and production patterns |
| Goal 13. | Take urgent action to combat climate change and its impacts |
| Goal 14. | Conserve and sustainably use the oceans, seas and marine resources for sustainable development |
| Goal 15. | Protect, restore and promote sustainable use of terrestrial ecosystems, sustainably manage forests, combat desertification, and halt and reverse land degradation and halt biodiversity loss |
| Goal 16. | Promote peaceful and inclusive societies for sustainable development, provide access to justice for all and build effective, accountable and inclusive institutions at all levels |
| Goal 17. | Strengthen the means of implementation and revitalise the Global Partnership for Sustainable Development |

**S2 Appendix. Applied style descriptors & factors, calculation methods, Bloomberg codes**

| **No** | **Factor** | **No** | **Descriptor** | **Brief description of the calculation methods** | **Bloomberg codes applied for calculation** |
| --- | --- | --- | --- | --- | --- |
| 1 | Beta (B) | 1 | Beta-1 | Market-relative beta. Regression on total returns (explanatory variable is the total return of the market; the dependent variable is the total return of the given share). The reference period is one year. | TOT_RETURN_INDEX_NET_DVDS |
| 2 | Value (V) | 2 | E/P | The inverse of forward P/E. The EPS is for the next four upcoming quarters. | BEST_PE_RATIO |
|  |  | 3 | CF/P | The inverse of P/CF. The Cash Flow Per Share is calculated on a trailing 12-month basis where available. | PX_TO_CASH_FLOW |
|  |  | 4 | BV/P | The inverse of P/BV. The BV data are from the most recent reporting period (quarterly, semi-annual or annual). | PX_TO_BOOK_RATIO |
| 3 | Momentum (M) | 5 | Return momentum | The sum of the weekly total returns for a given day (reference period: one year; from t-56 to t-4), excluding the last 4 weeks due to the reversal effect. | TOT_RETURN_INDEX_NET_DVDS |
|  |  | 6 | Price momentum | The stock price for a given day divided by the highest price of the last year (last 4 weeks are excluded due to the reversal effect; from t-56 to t-4). | PX_LAST |
|  |  | 7 | Sharpe-momentum | The sum of the weekly total returns for a given day for the last year, excluding the last 4 weeks due to the reversal effect (from t-56 to t-4). This is divided by the standard deviation (SD) of the weekly total returns of the last year (last four weeks also are excluded). SD is annualised. | TOT_RETURN_INDEX_NET_DVDS |
| 4 | Size (S) | 8 | -ln(MCap) | These are measures for the small size effect. | CUR_MKT_CAP |
|  |  | 9 | -ln(Assets) |  | BS_TOT_ASSET |
|  |  | 10 | -ln(Sales) |  | SALES_REV_TURN |
| 5 | Volatility (Vol) | 11 | Total volatility | Return volatility over latest 252 trading days. | PX_LAST |
|  |  | 12 | Residual volatility | Residual volatility: total volatility - (beta x volatility of the market). |  |
|  |  | 13 | Price range | The ratio of maximum and minimum stock price over the previous year. |  |
| 6 | Liquidity (L) | 14 | Amihud ratio | The inverse of the Amihud ratio. | VOLUME, PX_LAST |
| 7 | Profitability (P) | 15 | ROE | Return on common equity | RETURN_COM_EQY |
|  |  | 16 | ROA | Return on assets | RETURN_ON_ASSET |
|  |  | 17 | ROIC/WACC | Return on invested capital/Weighted average cost of capital | ROC_WACC_RATIO |
|  |  | 18 | ROS | Return on sales (profit margin, net income/sales) | PROF_MARGIN |

*(Continued)*

| **No** | **Factor** | **No** | **Descriptor** | **Calculation method** | **Bloomberg codes applied for calculation** |
| --- | --- | --- | --- | --- | --- |
| 8 | Growth (G) | 19 | EBT growth | For a given day: the average increase in earnings before tax (EBT), sales and net income for the last four years in the numerator, average assets of the last four years in the denominator. | PRETAX_INC |
|  |  | 20 | Net income growth |  | IS_INC_BEF_XO_ITEM |
|  |  | 21 | Sales growth |  | SALES_REV_TURN |
| 9 | Investment (I) | 22 | Asset growth | For a given day: the average increase in assets for the last four years in the numerator, average assets of the last four years in the denominator. | BS_TOT_ASSET |
| 10 | Leverage (L) | 23 | Book leverage | In the numerator: long + short loans (the latter netted with cash). Denominator: equity book value, equity market value and assets. | BS_LT_BORROW, BS_ST_BORROW |
|  |  | 24 | Market leverage |  | BS_CASH_NEAR_CASH_ITEM |
|  |  | 25 | Debt/Assets |  | CUR_MKT_CAP, TOT_COMMON_EQY, BS_TOT_ASSET |
| 11 | Earnings variability (EV) | 26 | Sales variability | In the numerator: standard deviation of the net income, FCFF, Sales volatility for the last four years; in the denominator: the median total assets for the last four years. | CF_CASH_FROM_OPER, SALES_REV_TURN |
|  |  | 27 | Net income variability |  | IS_INC_BEF_XO_ITEM |
|  |  | 28 | FCFF variability |  | BS_TOT_ASSET |

Descriptors are merged via Principal Component Analysis (PCA).

**S3 Appendix. Missing data in the database**

| **Descriptor** | **Missing** | **Total** | **Per cent missing** |
| --- | --- | --- | --- |
| Beta | 4 155 | 542 906 | 0.77% |
| E/P | 8 080 | 542 906 | 1.49% |
| CF/P | 65 192 | 542 906 | 12.01% |
| BV/P | 15 082 | 542 906 | 2.78% |
| Return momentum | 52 | 542 906 | 0.01% |
| Price momentum | 99 | 542 906 | 0.02% |
| Sharpe-momentum | 147 | 542 906 | 0.03% |
| -ln(MCap), | 52 | 542 906 | 0.01% |
| -ln(Assets), | 1 049 | 542 906 | 0.19% |
| -ln(Sales) | 15 157 | 542 906 | 2.79% |
| Total volatility | 79 | 542 906 | 0.01% |
| Residual volatility | 4 155 | 542 906 | 0.77% |
| Price range | 57 | 542 906 | 0.01% |
| Amihud | 4 104 | 542 906 | 0.76% |
| ROE | 6 530 | 542 906 | 1.20% |
| ROA | 2 697 | 542 906 | 0.50% |
| ROIC/WACC | 1 049 | 542 906 | 0.19% |
| Profit margin | 1 540 | 542 906 | 0.28% |
| Asset growth | 30 133 | 542 906 | 5.55% |
| Net income growth | 8 137 | 542 906 | 1.50% |
| Sales growth | 30 032 | 542 906 | 5.53% |
| Book leverage | 3 209 | 542 906 | 0.59% |
| Market leverage | 3 209 | 542 906 | 0.59% |
| Debts/Assets. | 3 209 | 542 906 | 0.59% |
| Sales variability | 20 273 | 542 906 | 3.73% |
| Net income variability | 17 525 | 542 906 | 3.23% |
| FCFF variability | 19 753 | 542 906 | 3.64% |
| **Total** | **264 756** | **14 658 464** | **1.81%** |

**S4 Appendix. Industry and country classification**

| **№** | **Industry groups (second level GICS)** |  | **Countries** | | | |
| --- | --- | --- | --- | --- | --- | --- |
| 1 | Automobiles & Components |  | 1 | United Arab Emirates | 25 | India |
| 2 | Banks |  | 2 | Austria | 26 | Italy |
| 3 | Capital Goods |  | 3 | Australia | 27 | Japan |
| 4 | Commercial & Professional Services |  | 4 | Belgium | 28 | Korea, Republic of |
| 5 | Consumer Durables & Apparel |  | 5 | Bermuda | 29 | Liberia |
| 6 | Consumer Services |  | 6 | Brazil | 30 | Luxembourg |
| 7 | Diversified Financials |  | 7 | Canada | 31 | Mexico |
| 8 | Energy |  | 8 | Switzerland | 32 | Malaysia |
| 9 | Food & Staples Retailing |  | 9 | Chile | 33 | Netherlands |
| 10 | Food, Beverage & Tobacco |  | 10 | China | 34 | Norway |
| 11 | Health Care Equipment & Services |  | 11 | Colombia | 35 | New Zealand |
| 12 | Household & Personal Products |  | 12 | Curacao | 36 | Peru |
| 13 | Insurance |  | 13 | Czech Republic | 37 | Philippines |
| 14 | Materials |  | 14 | Germany | 38 | Poland |
| 15 | Media & Entertainment |  | 15 | Denmark | 39 | Portugal |
| 16 | Pharmaceuticals, Biotechnology & Life Sciences |  | 16 | Spain | 40 | Qatar |
| 17 | Real Estate |  | 17 | Finland | 41 | Russian Federation |
| 18 | Retailing |  | 18 | France | 42 | Sweden |
| 19 | Semiconductors & Semiconductor Equipment |  | 19 | United Kingdom | 43 | Singapore |
| 20 | Software & Services |  | 20 | Greece | 44 | Thailand |
| 21 | Technology Hardware & Equipment |  | 21 | Hong Kong | 45 | Turkey |
| 22 | Telecommunication Services |  | 22 | Hungary | 46 | Taiwan |
| 23 | Transportation |  | 23 | Ireland | 47 | United States |
| 24 | Utilities |  | 24 | Israel | 48 | South Africa |

**S5 Appendix. Thematic ETFs, Assets Under Management (AUM), $ million, 27.09.2019.**

| **№** | **ETF Ticker** | **ETF Name** | **Megatrend group** | **Megatrend** | **AUM** |
| --- | --- | --- | --- | --- | --- |
| 1 | ETHO US Equity | ETHO CLIMATE LEADERSHIP ETF | Environmental | Energy Efficiency | 53 |
| 2 | ACES US Equity | ALPS CLEAN ENERGY ETF | Environmental | Energy Efficiency | 88 |
| 3 | HAP US Equity | VANECK NATURAL RESOURCES | Environmental | Energy Efficiency | 65 |
| 4 | INRG LN Equity | ISHARES GLOBAL CLEAN ENERGY | Environmental | Energy Efficiency | 288 |
| 5 | PBD US Equity | INVESCO GLOBAL CLEAN ENERGY | Environmental | Energy Efficiency | 48 |
| 6 | PBW US Equity | INVESCO WILDERHILL CLEAN ENE | Environmental | Energy Efficiency | 200 |
| 7 | PZD US Equity | INVESCO CLEANTECH ETF | Environmental | Energy Efficiency | 193 |
| 8 | COW CN Equity | ISHARES GLOBAL AGRICULTURE I | Environmental | Food Security | 177 |
| 9 | ISAG LN Equity | ISHARES AGRIBUSINESS | Environmental | Food Security | 61 |
| 10 | MOO US Equity | VANECK AGRIBUSINESS | Environmental | Food Security | 656 |
| 11 | CGW US Equity | INVESCO S&P GLOBAL WATER IND | Environmental | Water Scarcity | 669 |
| 12 | CWW CN Equity | ISHARES GLOBAL WATER INDEX E | Environmental | Water Scarcity | 125 |
| 13 | FIW US Equity | FIRST TRUST WATER ETF | Environmental | Water Scarcity | 468 |
| 14 | PHO US Equity | INVESCO WATER RESOURCES ETF | Environmental | Water Scarcity | 1 012 |
| 15 | PIO US Equity | INVESCO GLOBAL WATER ETF | Environmental | Water Scarcity | 188 |
| 16 | AGED LN Equity | ISHARES AGEING POPULATION | Social | Ageing | 255 |
| 17 | MILN US Equity | GLOBAL X MILLENNIALS THEMATIC | Social | Millennials | 76 |
| 18 | CIF CN Equity | ISHARES GLOBAL INFRASTRUCTURE | Social | Urbanisation | 111 |
| 19 | GII US Equity | SPDR S&P GLOBAL INFRASTRUCTURE | Social | Urbanisation | 400 |
| 20 | IGF US Equity | ISHARES GLOBAL INFRASTRUCTURE | Social | Urbanisation | 3 258 |
| 21 | MICH AU Equity | MAGELLAN INFRA FUND-CURR HGD | Social | Urbanisation | 303 |
| 22 | NFRA US Equity | FLEXSHARES STOXX GLOBAL BROA | Social | Urbanisation | 1 421 |
| 23 | PAVE US Equity | GLOBAL X US INFRASTRUCTURE | Social | Urbanisation | 135 |
| 24 | QIF CN Equity | AGFIQ ENHANCED GLOBAL INFRA | Social | Urbanisation | 229 |
| 25 | TOLZ US Equity | PROSHARES GLB INFRASTRUCTURE | Social | Urbanisation | 113 |
| 26 | XSGI GR Equity | X S&P GLOBAL INFRA SWAP | Social | Urbanisation | 226 |
| 27 | ZGI CN Equity | BMO GLOBAL INFRASTRUCTURE | Social | Urbanisation | 183 |
| 28 | BLCN US Equity | REALITY SHRS NASDAQ NEXGEN | Governance | Disruptive Technology | 66 |
| 29 | CYBR CN Equity | EVOLVE CYBERSECURITY INDEX | Governance | Cybersecurity | 44 |
| 30 | HACK AU Equity | BETASHARES GLOBAL CYBERSECURITY | Governance | Cybersecurity | 103 |
| 31 | BOTZ US Equity | GLOBAL X ROBOTICS & ARTIFICI | Governance | Robotics | 1 420 |
| 32 | IRBO US Equity | ISHARES ROBOTICS & ARTIFICIAL | Governance | Robotics | 49 |
| 33 | RBOT LN Equity | ISHARES AUTOMATION&ROBOTIC-A | Governance | Robotics | 1 999 |
| 34 | ROAI LN Equity | LYXOR ROBOTICS & AI ETF | Governance | Robotics | 116 |
| 35 | ROBO LN Equity | L&G ROBO GLOBAL ROBOTICS&AUT | Governance | Robotics | 862 |
| 36 | ROBO US Equity | ROBO GLOBAL ROBOTICS AND AUT | Governance | Robotics | 1 220 |
| 37 | ROBT US Equity | FIRST TRUST NASDAQ ARTIFICIAL | Governance | Robotics | 62 |
| **Total AUM** | | | | | **16 943** |

**S6 Appendix. Tests of robust instrumental variables**

To determine the validity of the GMM-IV_d_ estimator, one should calculate some tests. Based on [1], we apply a relevance test, an exogeneity test and a modified *Hausman* [2] artificial regression test (Hausman_d_). The relevance test is to check the robustness of the IVs. Relevance test helps in deciding if weak instruments happen to be in the analysis. According to *Racicot and Rentz* [3], an instrument is weak when it is only slightly correlated with the explanatory endogenous variables. The authors apply the method developed by *Olea and Pflueger* [4] who argue if the resulting F-statistics from regressions of the explanatory variables on the IVs are smaller than 24 for all of the regressions is an indication of a potential weak instruments problem. If at least one of the F values is above the critical value of 24, then the instruments are robust. The exogeneity test is the regression of residuals on the IVs. The residuals are estimated from equations (18)-(21) on every single megatrend. If the instruments are uncorrelated with the error terms, the instruments are exogenous. The Hausman_d_ is employed to check on measurement/specification errors (for detailed mathematical background see [5]).

Below, we present our results based on Fama-French 5-factor model; however, the conclusions are representative in each model specification and in every case of exogeneity test for all the megatrends.

Firstly, we present the relevance test. Each F-statistic is well above the critical 24, i.e. the instruments are robust. The diagonal elements are very close to 1 and have high t statistics, which means that each instrument is highly related to its respective explanatory variable.

**Table S6.1. Relevance test of robust instruments**

|  | **d_1_** | **d_2_** | **d_3_** | **d_4_** | **d_5_** | **c** | **F** |
| --- | --- | --- | --- | --- | --- | --- | --- |
| MRP | 1.000 | 0.000 | 0.000 | 0.000 | 0.000 | 0.001 | 131.05 |
| t | *24.354* | *0.000* | *0.000* | *0.000* | *0.000* | *2.128* |  |
| SIZE | 0.000 | 1.000 | 0.000 | 0.000 | 0.000 | 0.000 | 306.47 |
| t | *0.000* | *36.107* | *0.000* | *0.000* | *0.000* | *2.855* |  |
| VALUE | 0.000 | 0.000 | 1.000 | 0.000 | 0.000 | 0.000 | 455.26 |
| t | *0.000* | *0.000* | *41.811* | *0.000* | *0.000* | *3.689* |  |
| PROFIT | 0.000 | 0.000 | 0.000 | 1.000 | 0.000 | 0.000 | 326.18 |
| t | *0.000* | *0.000* | *0.000* | *37.060* | *0.000* | *6.325* |  |
| INV | 0.000 | 0.000 | 0.000 | 0.000 | 1.000 | 0.000 | 441.28 |
| t | *0.000* | *0.000* | *0.000* | *0.000* | *41.867* | *4.139* |  |

Next, the following table summarises the exogeneity test, and we introduce the case of water scarcity megatrend. Each coefficient of the instrumental variables is close to 0; further, they are not significant as their p-values are higher than any of the usual significance levels. Besides, the R^2^ is very close to 0. Thus, we conclude that our instruments are exogenous.

**Table S6.2. Exogeneity test for robust instruments**

|  | **d_1_** | **d_2_** | **d_3_** | **d_4_** | **d_5_** | **c** |
| --- | --- | --- | --- | --- | --- | --- |
| Coef | -0.0029 | 0.0069 | 0.0093 | -0.0148 | -0.0155 | 0.0000 |
| p-value | 0.433 | 0.724 | 0.725 | 0.624 | 0.626 | 1.000 |
| R2 | 0.0058 |  |  |  |  |  |

Finally, the table below presents the output of Hausman_d_ artificial regression tests. (The table repeat the regression coefficients of the GMM-IV_d_ method from Table 5. The coefficients of the explanatory variables are the same in the two approaches, which corresponds to what is expected.) The t statistics of ω’s are mostly insignificant, indicating the lack of measurement errors. However, the F tests confirm the presence of errors-in-variables in the case of 5 megatrends.

**Table S6.3. Hausman_d_ estimation method of the Fama-French 5-factor model**

|  | **c** | **MRP** | **SIZE** | **VALUE** | **PROFIT** | **INV** | **ω_MRP_** | **ω_SIZE_** | **ω_VALUE_** | **ω_PROFIT_** | **ω_INV_** |
| --- | --- | --- | --- | --- | --- | --- | --- | --- | --- | --- | --- |
| ***Energy efficiency*** | | | | | | | | | | | |
| Coef. GMM | 0.000 | 0.994 | 0.012 | 0.057 | 0.157 | 0.332 |  |  |  |  |  |
| Coef | 0.000 | 0.994 | 0.012 | 0.057 | 0.157 | 0.332 | 0.010 | -0.007 | -0.041 | 0.150 | 0.174 |
| SE | 0.000 | 0.005 | 0.024 | 0.032 | 0.037 | 0.039 | 0.014 | 0.095 | 0.122 | 0.159 | 0.213 |
| t | 0.140 | 216.671 | 0.507 | 1.770 | 4.242 | 8.524 | 0.723 | -0.069 | -0.337 | 0.946 | 0.820 |
| p-value | 0.889 | 0.000 | 0.612 | 0.078 | 0.000 | 0.000 | 0.471 | 0.945 | 0.736 | 0.345 | 0.413 |
|  |  |  |  |  |  | F-test | 1.540 | | | | |
|  |  |  |  |  |  | p-value | 0.177 | | | | |
| ***Food security*** | | | | | | | | | | | |
| Coef. GMM | 0.000 | 0.991 | 0.034 | 0.107 | 0.149 | 0.310 |  |  |  |  |  |
| Coef | 0.000 | 0.991 | 0.034 | 0.107 | 0.149 | 0.310 | 0.015 | -0.048 | -0.135 | 0.173 | 0.179 |
| SE | 0.000 | 0.004 | 0.019 | 0.026 | 0.030 | 0.031 | 0.011 | 0.076 | 0.098 | 0.128 | 0.171 |
| t | 0.043 | 267.809 | 1.741 | 4.118 | 5.000 | 9.856 | 1.332 | -0.634 | -1.377 | 1.347 | 1.044 |
| p-value | 0.966 | 0.000 | 0.083 | 0.000 | 0.000 | 0.000 | 0.184 | 0.526 | 0.170 | 0.179 | 0.297 |
|  |  |  |  |  |  | F-test | 2.800 | | | | |
|  |  |  |  |  |  | p-value | 0.018** | | | | |
| ***Water scarcity*** | | | | | | | | | | | |
| Coef. GMM | 0.000 | 0.996 | 0.040 | 0.063 | 0.155 | 0.347 |  |  |  |  |  |
| Coef | 0.000 | 0.996 | 0.040 | 0.063 | 0.155 | 0.347 | 0.006 | -0.098 | -0.099 | 0.061 | 0.212 |
| SE | 0.000 | 0.004 | 0.021 | 0.029 | 0.033 | 0.035 | 0.012 | 0.085 | 0.109 | 0.142 | 0.190 |
| t | 0.808 | 243.069 | 1.867 | 2.186 | 4.701 | 9.987 | 0.534 | -1.163 | -0.911 | 0.431 | 1.119 |
| p-value | 0.420 | 0.000 | 0.063 | 0.030 | 0.000 | 0.000 | 0.594 | 0.246 | 0.363 | 0.667 | 0.264 |
|  |  |  |  |  |  | F-test | 1.250 | | | | |
|  |  |  |  |  |  | p-value | 0.2854 | | | | |
| ***Aging*** | | | | | | | | | | | |
| Coef. GMM | 0.000 | 0.988 | 0.042 | 0.123 | 0.135 | 0.295 |  |  |  |  |  |
| Coef | 0.000 | 0.988 | 0.042 | 0.123 | 0.135 | 0.295 | 0.018 | -0.067 | -0.281 | 0.213 | 0.359 |
| SE | 0.000 | 0.005 | 0.028 | 0.038 | 0.044 | 0.046 | 0.016 | 0.112 | 0.144 | 0.187 | 0.250 |
| t | -0.944 | 182.868 | 1.478 | 3.240 | 3.107 | 6.428 | 1.159 | -0.599 | -1.956 | 1.138 | 1.432 |
| p-value | 0.346 | 0.000 | 0.141 | 0.001 | 0.002 | 0.000 | 0.248 | 0.550 | 0.052* | 0.256 | 0.153 |
|  |  |  |  |  |  | F-test | 3.380 | | | | |
|  |  |  |  |  |  | p-value | 0.006*** | | | | |

*(Continued)*

|  | **c** | **MRP** | **SIZE** | **VALUE** | **PROFIT** | **INV** | **ω_MRP_** | **ω_SIZE_** | **ω_VALUE_** | **ω_PROFIT_** | **ω_INV_** |
| --- | --- | --- | --- | --- | --- | --- | --- | --- | --- | --- | --- |
| ***Millennials*** | | | | | | | | | | | |
| Coef. GMM | 0.000 | 0.996 | 0.026 | 0.056 | 0.173 | 0.364 |  |  |  |  |  |
| Coef | 0.000 | 0.996 | 0.026 | 0.056 | 0.173 | 0.364 | 0.008 | -0.080 | -0.079 | 0.005 | 0.242 |
| SE | 0.000 | 0.005 | 0.028 | 0.038 | 0.043 | 0.046 | 0.016 | 0.111 | 0.143 | 0.186 | 0.249 |
| t | -0.148 | 185.434 | 0.921 | 1.474 | 4.000 | 7.973 | 0.511 | -0.722 | -0.554 | 0.025 | 0.971 |
| p-value | 0.883 | 0.000 | 0.358 | 0.142 | 0.000 | 0.000 | 0.610 | 0.471 | 0.580 | 0.980 | 0.333 |
|  |  |  |  |  |  | F-test | 0.500 | | | | |
|  |  |  |  |  |  | p-value | 0.778 | | | | |
| ***Urbanisation*** | | | | | | | | | | | |
| Coef. GMM | 0.000 | 0.988 | 0.055 | 0.024 | 0.173 | 0.454 |  |  |  |  |  |
| Coef | 0.000 | 0.988 | 0.055 | 0.024 | 0.173 | 0.454 | 0.011 | -0.037 | 0.020 | 0.245 | 0.022 |
| SE | 0.000 | 0.006 | 0.030 | 0.040 | 0.046 | 0.048 | 0.017 | 0.118 | 0.151 | 0.197 | 0.264 |
| t | 0.342 | 173.334 | 1.843 | 0.604 | 3.774 | 9.379 | 0.633 | -0.318 | 0.131 | 1.241 | 0.082 |
| p-value | 0.733 | 0.000 | 0.067 | 0.547 | 0.000 | 0.000 | 0.527 | 0.751 | 0.896 | 0.216 | 0.934 |
|  |  |  |  |  |  | F-test | 1.220 | | | | |
|  |  |  |  |  |  | p-value | 0.302 | | | | |
| ***Cybersecurity*** | | | | | | | | | | | |
| Coef. GMM | 0.000 | 0.985 | 0.037 | 0.102 | 0.107 | 0.334 |  |  |  |  |  |
| Coef | 0.000 | 0.985 | 0.037 | 0.102 | 0.107 | 0.334 | 0.019 | -0.088 | -0.087 | 0.166 | 0.024 |
| SE | 0.000 | 0.005 | 0.024 | 0.032 | 0.036 | 0.038 | 0.013 | 0.093 | 0.120 | 0.156 | 0.209 |
| t | -0.341 | 218.561 | 1.549 | 3.228 | 2.958 | 8.740 | 1.443 | -0.945 | -0.728 | 1.063 | 0.116 |
| p-value | 0.734 | 0.000 | 0.123 | 0.001 | 0.003 | 0.000 | 0.150 | 0.346 | 0.467 | 0.289 | 0.908 |
|  |  |  |  |  |  | F-test | 2.540 | | | | |
|  |  |  |  |  |  | p-value | 0.029** | | | | |
| ***DisruptiveTechnology*** | | | | | | | | | | | |
| Coef. GMM | 0.000 | 0.990 | 0.032 | 0.130 | 0.108 | 0.346 |  |  |  |  |  |
| Coef | 0.000 | 0.990 | 0.032 | 0.130 | 0.108 | 0.346 | 0.026 | 0.006 | -0.035 | 0.203 | -0.018 |
| SE | 0.000 | 0.004 | 0.022 | 0.030 | 0.034 | 0.036 | 0.013 | 0.088 | 0.113 | 0.147 | 0.197 |
| t | 0.391 | 233.112 | 1.431 | 4.354 | 3.147 | 9.591 | 2.066 | 0.074 | -0.313 | 1.381 | -0.093 |
| p-value | 0.696 | 0.000 | 0.154 | 0.000 | 0.002 | 0.000 | 0.040** | 0.941 | 0.755 | 0.169 | 0.926 |
|  |  |  |  |  |  | F-test | 2.310 | | | | |
|  |  |  |  |  |  | p-value | 0.045** | | | | |
| ***Robotics*** | | | | | | | | | | | |
| Coef. GMM | 0.000 | 1.002 | 0.029 | 0.118 | 0.146 | 0.344 |  |  |  |  |  |
| Coef | 0.000 | 1.002 | 0.029 | 0.118 | 0.146 | 0.344 | 0.019 | -0.079 | -0.279 | 0.186 | 0.048 |
| SE | 0.000 | 0.005 | 0.029 | 0.039 | 0.044 | 0.047 | 0.016 | 0.113 | 0.146 | 0.190 | 0.255 |
| t | -0.242 | 182.481 | 1.011 | 3.052 | 3.293 | 7.387 | 1.144 | -0.693 | -1.915 | 0.979 | 0.187 |
| p-value | 0.809 | 0.000 | 0.313 | 0.003 | 0.001 | 0.000 | 0.254 | 0.489 | 0.057* | 0.329 | 0.852 |
|  |  |  |  |  |  | F-test | 2.090 | | | | |
|  |  |  |  |  |  | p-value | 0.068* | | | | |
| Notes: *** p < 0.01 ** p < 0.05 * p < 0.10 | | | | | | | | | | | |

**References**

1. Racicot F-E, Rentz WF, Kahl A, Mesly O. Examining the dynamics of illiquidity risks within the phases of the business cycle. Borsa Istanbul Review. 2019;19: 117–131. doi:10.1016/j.bir.2018.12.001

2. Hausman JA. Specification Tests in Econometrics. Econometrica. 1978;46: 1251–1271. doi:10.2307/1913827

3. Racicot F-É, Rentz WF, Théoret R. Testing the new Fama and French factors with illiquidity: A panel data investigation. Finance. 2018;Vol. 39: 45–102. doi:10.3917/fina.393.0045

4. Olea JLM, Pflueger C. A Robust Test for Weak Instruments. Journal of Business & Economic Statistics. 2013;31: 358–369. doi:10.1080/00401706.2013.806694

5. Racicot F-E, Rentz WF. The Pástor-Stambaugh empirical model revisited: Evidence from robust instruments. J Asset Manag. 2015;16: 329–341. doi:10.1057/jam.2015.22
